# Working correlates of protection predict SchuS4-derived-vaccine candidates with improved efficacy against an intracellular bacterium, *Francisella tularensis*

**DOI:** 10.1038/s41541-022-00506-9

**Published:** 2022-08-17

**Authors:** Roberto De Pascalis, Blake Frey, Helen M. Rice, Varunika Bhargava, Terry H. Wu, Ross L. Peterson, J. Wayne Conlan, Anders Sjöstedt, Karen L. Elkins

**Affiliations:** 1 Laboratory of Mucosal Pathogens and Cellular Immunology, Division of Bacterial, Parasitic and Allergenic Products, Center for Biologics Evaluation and Research, U.S. Food and Drug Administration, Silver Spring, MD USA; 2 Center for Infectious Disease and Immunity and Department of Internal Medicine, University of New Mexico, Albuquerque, NM USA; 3 Vaccine Evaluation Branch, Division of Biostatistics, Office of Biostatistics and Epidemiology, Center for Biologics Evaluation and Research, U.S. Food and Drug Administration, Silver Spring, MD USA; 4 National Research Council Canada, Institute for Biological Sciences Ottawa, Ottawa, ON Canada; 5 Department of Microbiology, National Defense Research Establishment, Umeå, Sweden; 6 Present Address: University of Alabama-Birmingham, Birmingham, AL USA; 7 Present Address: University of Bristol, Bristol, UK; 8 Present Address: Regeneron Pharmaceuticals, Tarrytown, NY USA

**Keywords:** Bacterial host response, Lymphocyte activation, Live attenuated vaccines

## Abstract

*Francisella tularensis*, the causative agent of tularemia, is classified as Tier 1 Select Agent with bioterrorism potential. The efficacy of the only available vaccine, LVS, is uncertain and it is not licensed in the U.S. Previously, by using an approach generally applicable to intracellular pathogens, we identified working correlates that predict successful vaccination in rodents. Here, we applied these correlates to evaluate a panel of SchuS4-derived live attenuated vaccines, namely SchuS4-Δ*clpB*, *ΔclpB*-*ΔfupA*, *ΔclpB-ΔcapB*, and *ΔclpB-ΔwbtC*. We combined in vitro co-cultures to quantify rodent T-cell functions and multivariate regression analyses to predict relative vaccine strength. The predictions were tested by rat vaccination and challenge studies, which demonstrated a clear relationship between the hierarchy of in vitro measurements and in vivo vaccine protection. Thus, these studies demonstrated the potential power a panel of correlates to screen and predict the efficacy of *Francisella* vaccine candidates, and in vivo studies in Fischer 344 rats confirmed that SchuS4*-*Δ*clpB* and *ΔclpB-ΔcapB* may be better vaccine candidates than LVS.

## Introduction

To determine efficacy with adequate power, clinical trials of new vaccine candidates are conducted with large numbers of people in areas where natural exposure to the microorganism in question occurs at a rate sufficient to detect vaccination outcomes. However, the low incidence of some infectious diseases makes this approach impractical for evaluating vaccine candidates against them. With a total number of 100–300 cases per year in the U.S., tularemia falls into this category^1^. Tularemia is caused by two subspecies of *Francisella tularensis* (*Ft*), the highly virulent Type A *F. tularensis* subsp. *tularensis* that is prevalent in North America, as well as Type B *F. tularensis* subsp. *holarctica* that is common in Eurasia^2^. Tularemia does not represent a public health problem in the U.S. because of its low incidence and because antibiotics are effective when the disease is diagnosed promptly. Nonetheless, a preventive vaccine is still of interest: due to its virulence and ability to be weaponized, *Ft* has been classified as Tier 1 Select Agent and is considered a potential bioweapon (https://www.selectagents.gov/).

Like many intracellular bacteria, vaccines against *Ft* have been difficult to develop. In the 1950’s, an attenuated vaccine dubbed Live Vaccine Strain (LVS) was developed from Type B *F. tularensis*^3,4^. LVS represents the most advanced tularemia vaccine candidate in western countries but is still investigational in the U.S. Remarkably, human challenge studies that exposed naive and LVS-vaccinated people to virulent Type A tularemia were performed in the 1960’s, during military-sponsored studies known as Operation Whitecoat^5^. These studies suggested LVS can provide partial protection in an experimental setting^6–8^, but its efficacy has not been evaluated in field trials. Challenge studies would likely be considered unethical now and would be limited by the number of volunteers that could be studied.

*Ft* infects a wide variety of animals and humans, replicating primarily in macrophages^9,10^. In all mammals studied to date, *Ft* infection induces both humoral and T cell-mediated immune responses. Like many intracellular bacteria and parasites (here, intracellular pathogens), *Ft*-specific T-cell functions likely dominate protection against re-infection as well as protection induced by live attenuated vaccines^11,12^, and studies of *Francisella* immunity shed light on immunity to intracellular bacteria in general^13^. We and others have used both inbred mice and Fischer 344 rats to study immunity to *Francisella* because both these models have similarities to human infections but have different advantages^14–16^. While mice are useful for screening novel vaccines and particularly for mechanistic studies, Fischer 344 rats have important additional similarities to human *Francisella* infections. Unlike mice, rats survive moderate doses of infection with Type B *Ft* strains and with *F. novicida*, another *Francisella* species that rarely causes human disease^17^. Moreover, rats vaccinated subcutaneously (s.c.) with LVS survive large intratracheal (i.t.) or aerosol challenge doses with highly virulent Type A *Ft* SchuS4^18^.

Although the incidence of tularemia in some regions of Europe and Scandinavia is relatively high^19^, the prevalence of Type B *Ft* in those regions limits the evaluation of the efficacy of tularemia vaccines against Type A *Ft*. When human efficacy trials are not feasible, animal studies may be the best option to screen and evaluate new vaccine candidates. This approach, including evaluating vaccines under the FDA “Animal Rule”^20–22^, depends on developing a rational means to bridge the outcomes in animals to humans. Quantifying functional and relevant immune responses would provide the means to do so. However, no validated correlates of protection have been identified to date in humans for any intracellular pathogen. Thus, no correlate strategies are available to aid clinical studies of vaccines against these pathogens including those against tularemia. Ideally, meaningful correlates of vaccine-induced protection that can be measured across species should be developed. Because immunity to *Francisella* is similar to that against many other intracellular pathogens^13,23^, *Francisella* vaccine development offers an opportunity to evaluate strategies that may then be applied to vaccine development for other bacteria and parasites of public health importance^23^.

By focusing on cellular immune responses, we previously identified potential in vitro correlates of vaccine-induced protection against *Ft* in both mouse and rat models. We took advantage of a co-culture leukocyte re-stimulation approach that was designed to simulate the in vivo interactions between *Ft*, macrophages, and lymphocytes. In this approach, bone marrow-derived macrophages are infected in vitro with *Ft* LVS and co-cultured with *Francisella*-immune lymphocytes derived from vaccinated animals. LVS-infected macrophages stimulate *Ft*-immune T cells, which in turn control the intramacrophage growth of *Francisella* bacteria^11,12,24^. Importantly, we demonstrated that the relative ability of re-stimulated T cells to control the intramacrophage LVS replication directly reflects the relative efficacy of LVS-related vaccines^14,25–27^. In addition, by studying the lymphocytes recovered from re-stimulated co-cultures, we identified 14 genes whose relative expression also correlates with the in vivo efficacy of LVS-related vaccines.

Because LVS is imperfect, several next-generation *Francisella* vaccines have been developed^28–30^. These include live attenuated mutants of Type A *Ft* strain SchuS4 and mutants of *F. novicida*^29–31^. Some of these candidates have been tested in small animals, with mixed results, but none have been tested in humans. Among these, *Ft* Type A SchuS4-*ΔclpB* (*ΔclpB*) vaccine showed promising results in BALB/c mice but was less protective in C57BL/6 mice, potentially due to qualitatively different immune responses between inbred mouse strains^32^. To further reduce the potential of genetic reversion, a second gene, either *fupA*, *capB*, or *wbtC*, was deleted from *ΔclpB*, generating three double mutant vaccine candidates^33^. In our previous studies, we optimized methods to identify correlates of vaccine-induced protection by taking advantage of vaccines of known in vivo efficacy, all of which were derived from Type B *Ft*. In this study, we used those potential correlates to screen vaccines, all of which were derived from Type A *Ft*. With the exception of *ΔclpB*-*ΔwbtC*^34^, all have unknown in vivo efficacy in the rat model. In particular, we compared these candidate vaccines using the in vitro co-culture approach and the working panel of candidate genes to assess whether the potential correlates of protection could predict the in vivo efficacy of novel vaccines. We then performed vaccination and challenge studies in mice and in Fischer 344 rats and found a strong relationship between in vitro and in vivo outcomes. The results demonstrated that *ΔclpB* and *ΔclpB*-*ΔcapB* may be better vaccine candidates than LVS, which in turn is better than *ΔclpB*-*ΔwbtC* and *ΔclpB*-*ΔfupA*. The data therefore not only directly demonstrate the predictive power of this strategy and further support the value of the working correlates, but also indicate that *ΔclpB* and *ΔclpB*-*ΔcapB* merit advancement to higher animal model studies.

## Results

### *ΔclpB* vaccine induces strong in vitro T-cell immune responses in C57BL/6 mice

To compare *ΔclpB* to LVS-derived vaccines in a different animal model than BALB/c mice^32^, we vaccinated C57BL/6 mice intradermally (i.d.) with LVS, with the opacity variant LVS-R, used as a sub-optimal vaccine comparator^14,25,27,35^, or with *ΔclpB*. After 6 weeks, a subset of the mice was administered a lethal challenge of 1 × 10^6^ LVS intraperitoneally (i.p.). As expected, based on previous studies^25,27^, 30/31 mice (96.7%) vaccinated with LVS, and 12/22 mice vaccinated with LVS-R (54.5%) survived challenge. Further, 29/34 mice (85%) vaccinated with *ΔclpB* survived challenge. The remaining mice were used for in vitro co-culture studies. To evaluate the viability and composition of cells derived from vaccinated mice, splenocytes and peripheral blood lymphocytes (PBLs) added to co-cultures and then recovered from co-cultures after 48 h were characterized by flow cytometry. Analyses of the distribution of added and recovered leukocytes did not reveal any obvious differences in the viability or cellular composition between naive, *ΔclpB*-, and LVS-vaccinated mice, indicating that potential technical issues did not affect the results.

**Fig. 1:**
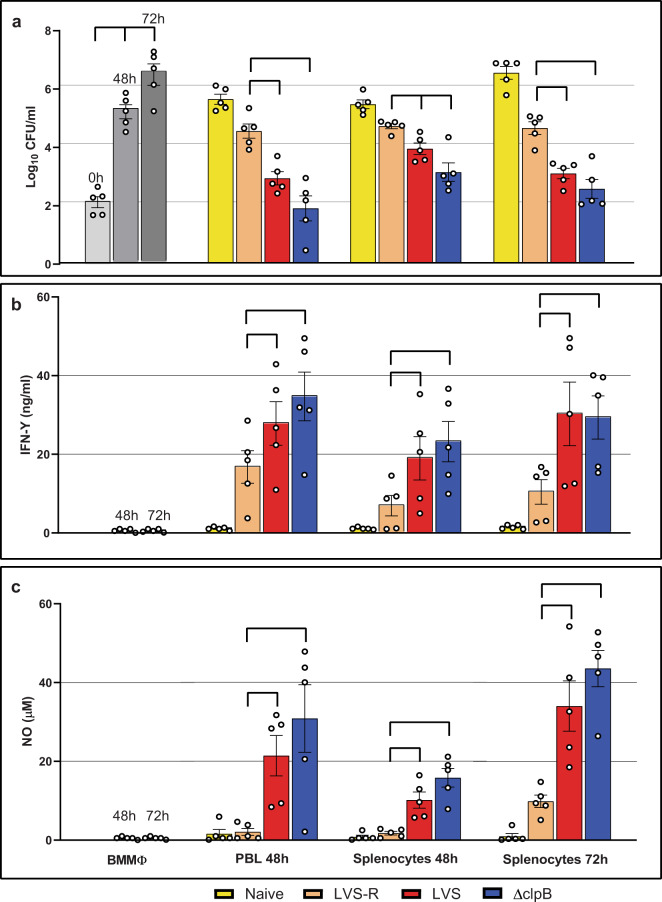
Leukocytes from *ΔclpB*-vaccinated mice control intramacrophage bacterial growth and effect mediator production in vitro. BMMΦ from C57BL/6J were infected with LVS and co-cultured with PBLs or splenocytes obtained from naive or vaccinated mice, as indicated. After two or three days of co-culture, BMMΦ were lysed to evaluate the recovery of intracellular bacteria (**a**). Supernatants from the corresponding co-cultures were collected and analyzed for IFN-γ (**b**) and NO (**c**). Values shown are the averages from five independent experiments of similar design. Error bars indicate the standard error of the mean (s.e.m.). Brackets indicate significant differences among vaccine groups, calculated by two-way ANOVA and Tukey’s multiple comparison test (*P* < 0.05). Symbols indicate values obtained from independent experiments. Data from naive samples were significantly different from the vaccine groups (not shown by brackets, for clarity), with the exception of NO results at 48 h, for which values from naive leukocytes were not significantly different from that of LVS-R.

Splenocytes and PBLs from *ΔclpB*-vaccinated mice were then used to evaluate their ability to control intramacrophage LVS replication, in comparison to leukocytes from LVS and LVS-R vaccinated mice (Fig. 1a). As previously demonstrated, leucocytes obtained from LVS-vaccinated mice efficiently controlled the intramacrophage growth of LVS. In contrast, leukocytes from LVS-R-vaccinated mice provided partial control and those from the naive mice provided no control compared to infected macrophages alone. Further, leukocytes from LVS-R-vaccinated mice or naive mice provided significantly less control compared those from either the LVS group or the *ΔclpB* group. PBLs and splenocytes from *ΔclpB*-vaccinated mice exhibited control of bacterial replication that was comparable to or, in three of five experiments, significantly better than that observed for LVS. Supernatants harvested from co-cultures were also evaluated for the production of IFN-γ (Fig. 1b) and nitric oxide (NO; Fig. 1c), both important mediators in responses to intracellular pathogens. Production of both mediators was significantly higher in the *ΔclpB* group compared to either the LVS-R group or the naive group. Across replicate experiments, production of both mediators trended higher in the *ΔclpB* group compared to those of the LVS group. Co-cultures containing cells from LVS-R vaccinated mice produced lower amounts of IFN-γ and minimal amounts of NO. Cell subpopulation separation studies were not practical here due to the limited number of PBLs available; however, the differences observed were likely due to T cell-mediated functions, because previous studies demonstrated that essentially all of the growth control activities of immune lymphocyte populations depend on T cells^26^.

The relative expression of 92 genes of immunologic interest was then tested in PBLs and splenocytes recovered after 2 days of co-culture. As previously demonstrated^14,27^, a number of genes were upregulated in PBLs from LVS-vaccinated mice compared to those from LVS-R-vaccinated mice (Table 1). When we compared the expression of these genes in PBLs derived from *ΔclpB*-vaccinated mice, three patterns emerged. The first pattern was comprised of genes with a comparable expression between cells from the *ΔclpB* and LVS groups (*ΔclpB* ≈ LVS > LVS-R). In the second and third patterns, the expression of other genes was consistently greater in cells from the *ΔclpB*-vaccinated group compared to that measured in cells from the LVS and LVS-R groups (*ΔclpB* > LVS > LVS-R, or *ΔclpB* > LVS ≈ LVS-R). However, in most cases, the differences were not significant. In general, relative gene expression in splenocytes followed similar patterns but was less pronounced.

**Table 1:** Relative gene expression of immune-related factors that correlate with protection in PBLs and splenocytes of C57BL/6 mice recovered from co-cultures at 48 h.

Gene	PBL			Splenocytes		
	*ΔclpB* ≈	LVS >	LVS-R	*ΔclpB*	LVS	LVS-R
IL-21	213 ± 48	198 ± 50*	40 ± 13.7*	76.1 ± 21.4	70.7 ± 25	19.4 ± 9.2
CCR5	22.0 ± 8.4	21.2 ± 9.9	10.9 ± 6.5	5.5 ± 1.2	5.0 ± 0.9*	2.8 ± 0.6*
Bcl-2	3.3 ± 0.6	4.8 ± 0.6*	2.4 ± 0.5*	1.7 ± 0.2	1.9 ± 0.2	1.6 ± 0.2
T-bet	4. 7 ± 1.2	3.9 ± 0.8*	1.9 ± 0.2*	3.0 ± 0.8	3.1 ± 0.4	2.2 ± 0.4
TNFRSF18	3.3 ± 0.5	4.1 ± 0.7	2.8 ± 0.7	1.5 ± 0.3	1.5 ± 0.1	1.5 ± 0.2
IL-2RA	4.8 ± 0.7	4.8 ± 0.7	3.8 ± 0.6	2.3 ± 0.9	2.1 ± 0.4	1.8 ± 0.4
C3	8.0 ± 4.1	7.1 ± 3.8	4.1 ± 1.3	6.1 ± 1.5	4.6 ± 0.8	4.3 ± 1.7
CXCL9	16.2 ± 6.7	19.4 ± 9.5	12.0 ± 5.0	9.7 ± 2.2	10.5 ± 3.0	7.5 ± 2.3
IFN-γ	24.8 ± 12	26.6 ± 16	10.7 ± 2.7	20.6 ± 12.6	10.9 ± 3.6	11.1 ± 2.1
HMOX1	4.2 ± 1.2	4.0 ± 1.8	1.4 ± 0.4	4.6 ± 1.4	3.2 ± 1.1	1.8 ± 0.6
	*ΔclpB* >	LVS >	LVS-R			
CCR2	19.9 ± 7.8	6.2 ± 2.9	3.3 ± 1.3	3.4 ± 1.1	3.0 ± 0.9	1.8 ± 0.5
CX3CR1	15.0 ± 4.9	5.8 ± 1.9*	1.3 ± 0.3*	18.8 ± 14.3	4.3 ± 2.3	1.4 ± 1.0
PRF1	16.9 ± 4.1	11.6 ± 2.8*	5.6 ± 1.4*	6.2 ± 1.7	4.8 ± 0.9	3.6 ± 0.6
NOS2	89.2 ± 66	44.4 ± 30	14.6 ± 6.9	32.8 ± 15.5	15.6 ± 5.5	9.8 ± 4.6
CCL5	6.7 ± 1.5	4.4 ± 1.0	3.0 ± 1.1	1.7 ± 0.2	1.6 ± 0.3	1.5 ± 0.3
CXCR6	6.1 ± 1.2*	3.2 ± 0.2*	2.5 ± 0.6	2.6 ± 0.8	2.1 ± 0.3*	1.4 ± 0.2*
LTA	10.5 ± 2.6	6.2 ± 1.2*	3.5 ± 0.6*	4.6 ± 1.1	3.2 ± 0.3	2.5 ± 0.4
GZMB	19.0 ± 5.1	15.1 ± 3.5	11.4 ± 2.7	7.8 ± 2.7	6.7 ± 1.3	6.6 ± 1.2
	*ΔclpB* >	LVS ≈	LVS-R			
CCL19	8.5 ± 2.3*	3.0 ± 0.9*	4.9 ± 2.1	1.4 ± 0.4	1.2 ± 0.4	1.2 ± 0.3
CCR1	4.7 ± 1.2*	1.3 ± 0.4*	1.4 ± 0.2	2.3 ± 0.4	1.5 ± 0.4	1.5 ± 0.4
PTGS2	13.8 ± 8.1	7.6 ± 4.2	6.7 ± 3.6	3.5 ± 0.8	2.5 ± 0.6	1.8 ± 0.4
VEGFA	3.7 ± 0.9	2.1 ± 0.4	1.9 ± 0.3	2.7 ± 0.3	2.1 ± 0.4	2.6 ± 0.4
IL-12rβ2	7.1 ± 2.0	4.9 ± 1.0	5.0 ± 1.3	3.8 ± 1.4	2.5 ± 0.5	2.6 ± 0.4
FASL	7.8 ± 2.3	4.8 ± 1.3	5.2 ± 1.8	3.3 ± 0.9	2.3 ± 0.2	2.9 ± 0.5
ICOS	3.3 ± 0.5*	2.1 ± 0.3*	2.0 ± 0.3	1.9 ± 0.5	1.4 ± 0.2	1.9 ± 0.5

Semiquantitative analyses of gene expression were performed using a custom array, as described in “Methods”. Fold changes were calculated in comparison to naïve cells. Data shown are mean of fold change ± standard error of the mean (s.e.m.) calculated from five independent experiments. Asterisks indicate significant differences in comparison to values observed for LVS-immune cells (*p* < 0.05).

A similar study of tuberculosis vaccines in mice used a whole-genome microarray to search for additional genes, identifying many that were not obviously related to immune functions^36^. We took advantage of this panel to explore the expression of 188 additional genes in PBLs recovered after two days of co-cultures. From this screen, we identified 30 genes that were differentially upregulated, with the general pattern of *ΔclpB* ≥ LVS > LVS-R (Table 2). Of these, 13 genes were among those that were upregulated twofold or more in PBLs from BCG-vaccinated mice^36^ compared to naive PBLs (Table 2, Group 1), while an additional 17 genes that were not differentially expressed in PBLs from BCG-vaccinated mice were differentially expressed between *ΔclpB*, LVS, and LVS-R (Table 2, Group 2).

**Table 2:** Relative gene expression of novel factors that correlate with protection in PBLs of C57BL/6 mice recovered from co-cultures at 48 h.

Gene	*Ft*-*ΔclpB*	LVS	LVS-R
Group 1			
* UBD*	11.5 ± 3.7	10.0 ± 5.9	3.3 ± 1.5
* SLC7A11*	7.9 ± 3.2	3.2 ± 1.1	1.1 ± 0.5
* SLAMF8*	5.2 ± 1.4	3.4 ± 1.1	1.9 ± 0.3
* CASP1*	2.0 ± 0.5	1.6 ± 0.5	1.5 ± 0.5
* SRXN1*	5.2 ± 1.7	2.8 ± 0.9	1.0 ± 0.2
* EXOC3L4*	4.3 ± 1.3	2.7 ± 0.6	2.2 ± 0.4
* CISH*	4.1 ± 1.0	4.0 ± 1.3	3.0 ± 0.8
* FPR2*	4.3 ± 1.6	3.0 ± 1.0	1.5 ± 0.2
* PLA2G7*	6.3 ± 1.9	4.2 ± 1.2*	1.4 ± 0.3*
* DRAM1*	4.1 ± 1.1	3.2 ± 0.9	1.9 ± 0.4
* RGS1*	4.6 ± 0.6	3.9 ± 0.5*	2.4 ± 0.4*
* MDM2*	2.3 ± 0.3	2.2 ± 0.2*	1.1 ± 0.2*
* GADD45G*	4.2 ± 0.8	5.0 ± 0.4*	2.4 ± 0.4*
Group 2			
* EGLN3*	21.8 ± 9.0	14.6 ± 5.8*	2.0 ± 0.7*
* CLEC4N*	13.3 ± 4.2	5.0 ± 1.3*	1.6 ± 0.3*
* DNMT3L*	7.9 ± 3.0	4.9 ± 2.9	1.6 ± 0.4
* CLEC4D*	4.1 ± 1.8	2.0 ± 0.6*	0.6 ± 0.1*
* ARG1*	53.8 ± 40.5	49.1 ± 46.2	3.0 ± 1.8
* SMOX*	8.8 ± 2.1*	4.1 ± 1.1*	2.6 ± 0.7
* CTSK*	7.4 ± 3.2	2.3 ± 0.5	1.4 ± 0.3
* HCAR2*	10.3 ± 5.3	5.9 ± 3.1	2.3 ± 0.6
* TLR13*	2.7 ± 0.6	1.5 ± 0.4	1.0 ± 0.2
* VIM*	5.6 ± 1.2	3.3 ± 0.7*	1.8 ± 0.2*
* FBLIM1*	3.4 ± 0.8*	1.5 ± 0.4*	1.0 ± 0.2
* IGF2BP2*	3.1 ± 0.6	2.0 ± 0.4	1.4 ± 0.2
* SCL11A1*	3.5 ± 0.9	2.3 ± 0.5	1.5 ± 0.3
* PIK3CB*	3.8 ± 1.0	1.6 ± 0.4	1.1 ± 0.2
* PPAP2B*	4.6 ± 1.9	2.3 ± 0.8	1.3 ± 0.4
* PHLDA3*	11.0 ± 7.5	11.5 ± 7.2	2.7 ± 1.3
* HILPDA*	4.8 ± 1.5	3.0 ± 0.8*	1.1 ± 0.2*

Semi-quantitative analyses of gene expression were performed using a custom array, as described in “Methods”. Fold changes were calculated in comparison to naive cells and the data shown are means of fold change and standard error of mean (s.e.m.) calculated from five independent experiments. Results indicate differentially expressed genes according to the hierarchy *Ft*-*ΔclpB* ≥ LVS > LVS-R. Group 1 includes those factors that were >twofold upregulated also in PBLs from mice vaccinated with BCG Pasteur and protected against *M. tuberculosis* challenge. Group 2 includes those factors that were screened but were not differentially expressed in PBLs from BCG-vaccinated mice. Asterisks indicate significant differences in comparison to values observed for LVS-immune cells (*P* < 0.05).

### *ΔclpB*-derived vaccines and LVS compared in Fischer 344 rats by using in vitro working correlates

**Fig. 2:**
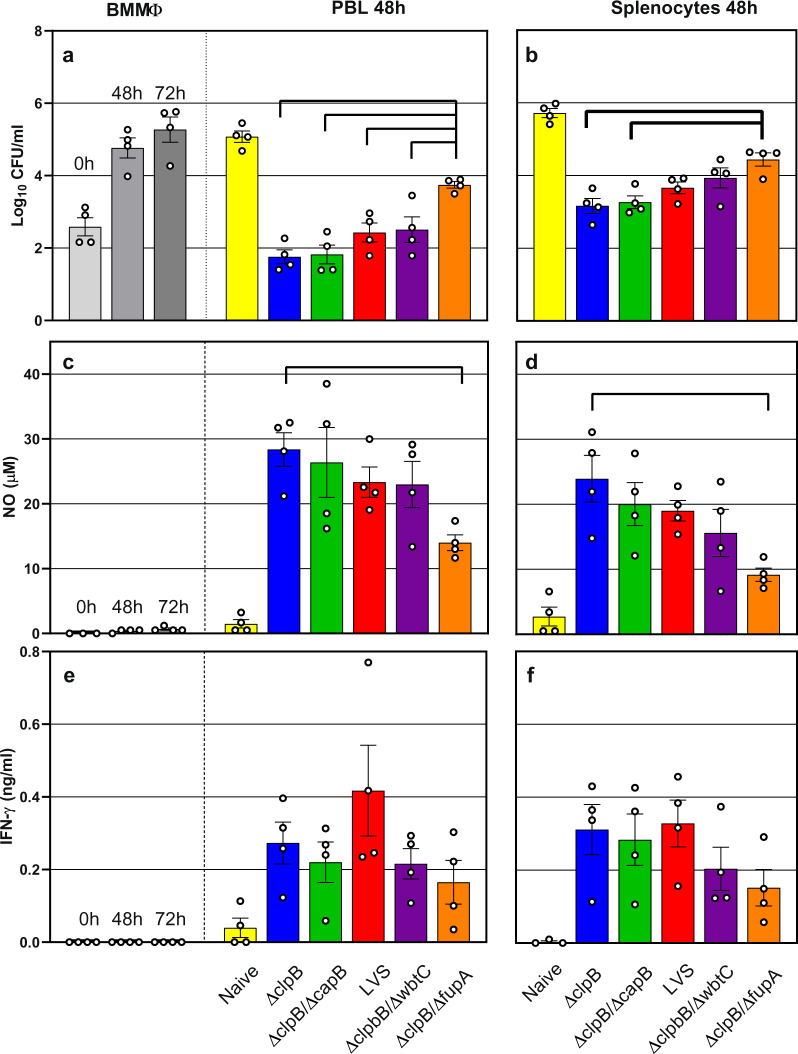
Rat leukocytes control intramacrophage bacterial growth and effect mediator production in vitro in a pattern that suggests differential vaccine efficacies. BMMΦ from Fischer 344 rats were infected with LVS and co-cultured with PBLs (**a**, **c**, **e**) or splenocytes (**b**, **d**, **f**) obtained from naive or vaccinated rats, as indicated. After 2 days of co-culture, BMMΦ were lysed to evaluate the recovery of intracellular bacteria (**a**, **b**). Supernatants were collected and analyzed for NO (**c**, **d**) and IFN-γ (**e**, **f**). Values shown are the average from four independent experiments of similar design. Error bars indicate standard error of the mean (s.e.m.). Brackets indicate significant differences among vaccine groups and calculated by two-way ANOVA and Tukey’s multiple comparison test (*P* < 0.05). Symbols indicate values obtained from independent experiments. Not shown by brackets are the differences calculated with naive samples against all vaccine groups, which were significant, except vs *ΔclpB*-*ΔfupA* (CFU, spleen; NO, PBLs at 48 h; NO, spleen), vs *ΔclpB*-*ΔcapB* (NO, spleen at 72 h), and vs *ΔclpB*-*ΔwbtC* (NO, spleen at 72 h).

We next evaluated *ΔclpB* vaccine and three other new *ΔclpB*-derived vaccines in Fischer 344 rats. Rats were vaccinated s.c. with *ΔclpB*, *ΔclpB*-*ΔfupA*, *ΔclpB-ΔcapB*, *ΔclpB-ΔwbtC*, or LVS. Similar to studies using mouse leukocytes, splenocytes and PBLs obtained from rats 6 weeks after vaccination were used to evaluate in vitro functions. PBLs obtained from *ΔclpB*-vaccinated rats controlled bacterial replication to a degree that was comparable to that found using PBLs from LVS-vaccinated rats (Fig. 2a), as seen in mouse experiments (Fig. 1a). Further, PBLs from *ΔclpB-ΔcapB*-vaccinated rats exhibited control of bacterial replication comparable to that of *ΔclpB*-vaccinated and LVS-vaccinated animals. In contrast, leukocytes from rats vaccinated with *ΔclpB-ΔwbtC* resulted in somewhat less control, and those from *ΔclpB*-*ΔfupA*-vaccinated rats provided significantly less control. A similar pattern was observed using splenocytes from naive or vaccinated rats (Fig. 2b). These patterns were consistent across experiments, but only control by leukocytes from *ΔclpB*-*ΔfupA*-vaccinated rats was significantly different from that provided by leukocytes from all other vaccination groups (see Fig. 2a).

To further characterize in vitro activities, we therefore analyzed levels of NO (Fig. 2c, d) and IFN-γ (Fig. 2e, f) in supernatants. Although most differences were not significant, levels of NO trended higher in supernatants from cultures containing leukocytes from *ΔclpB*- and *ΔclpB-ΔcapB*-vaccinated rats, followed by those from LVS- and *ΔclpB-ΔwbtC-*vaccinated rats, and finally by those from *ΔclpB*-*ΔfupA-*vaccinated animals. The pattern of higher NO production generally corresponded with co-cultures exhibiting lower bacterial growth (Fig. 2a, b). IFN-γ levels in supernatants from all co-cultures containing leukocytes from vaccinated animals were greater than those from naive animals, but similar to each other (Fig. 2e, f), and thus differences were less evident.

**Fig. 3:**
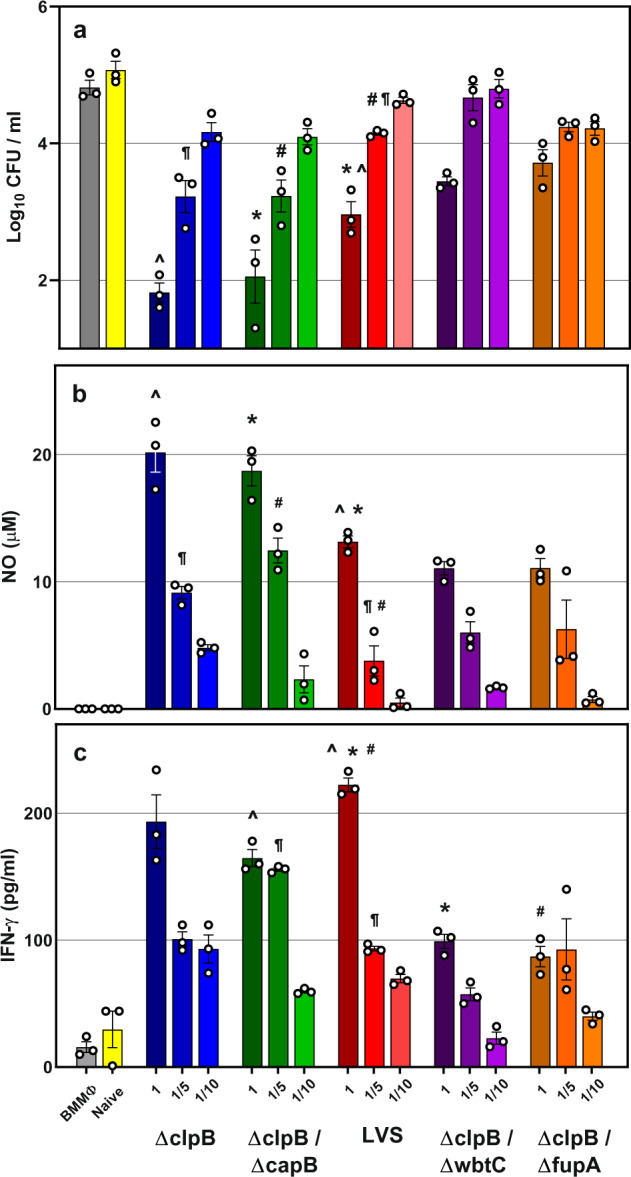
Titration of rat PBLs in co-cultures highlights a differential in vitro between vaccine groups. BMMΦ from Fischer rats were infected with LVS and co-cultured with decreasing amounts of PBLs obtained from naive or vaccinated rats, as indicated. After 2 days of co-culture, BMMΦ were lysed to evaluate the recovery of intracellular bacteria (**a**). Supernatants from the corresponding co-cultures were collected and analyzed for NO (**b**) and IFN-γ (**c**). Values shown are the average from samples run in triplicate; circle symbols indicate values for single data points. Error bars indicate the standard error of the mean (s.e.m.) (*P* < 0.05). Within each PBL dilution, differences were calculated by two-way ANOVA. Matching symbols indicate significant differences in comparison to LVS.

The degree of in vitro antibacterial activity is related to the number of *Ft*-specific T cells^14^. To further evaluate differences between vaccine groups, we performed co-cultures by titrating the amounts of added PBLs, using a tenfold range of cell numbers (Fig. 3). PBLs from the *ΔclpB*, *ΔclpB-ΔcapB*, and LVS-vaccinated groups exhibited control that was directly related to cell numbers. When using an intermediate number of PBLs (1/5 of that typically used, such as in Fig. 2), each group still partially controlled bacterial replication. In contrast, PBLs from *ΔclpB*-*ΔfupA* and *ΔclpB-ΔwbtC-*vaccinated rats exhibited less control. At the lowest number used (1/10), bacterial replication was poorly controlled by leukocytes from any of the vaccine groups. Overall, levels of NO (Fig. 3b) and IFN-γ (Fig. 3c) found in supernatants reflected the number of cells used in the co-cultures. Of note, the data set shown in Fig. 3, which is different from that shown in Fig. 2, is an example of an experiment in which control of intramacrophage growth by PBLs from *ΔclpB* and *ΔclpB-ΔcapB*-vaccinated rats is significantly different from that from LVS-immune PBLs.

**Fig. 4:**
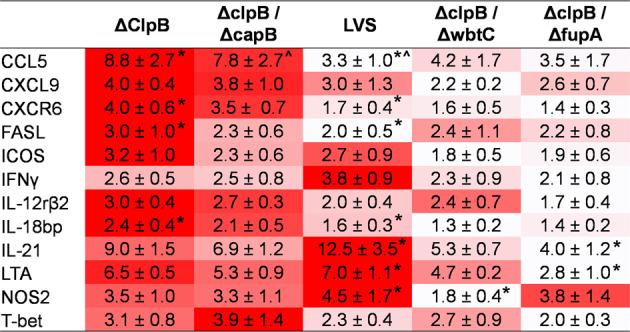
Relative gene expression of a working panel of correlates of protection in PBLs suggests a hierarchy of vaccine efficacy. BMMΦ from Fischer rats were infected with LVS and co-cultured with PBLs obtained from naive or vaccinated rats, as indicated. After 2 days of co-culture, PBLs were collected and analyzed for relative gene expression. Semiquantitative analyses of gene expression were performed using a custom array as described in “Methods”. Fold changes were calculated in comparison to naïve cells and data shown are averages of fold change ± standard error of the mean (s.e.m.) calculated from four independent experiments of a similar design. The heat map shown was generated within each gene across vaccine groups; darker colors indicate higher upregulation, while white indicates lower upregulation, within each gene. Matching symbols indicate significant differences in comparison to values observed for LVS-immune cells (*P* < 0.05).

To extend the evaluation of in vitro functions, PBLs and splenocytes recovered from co-cultures were analyzed for relative expression of genes of immunologic interest using a custom array containing a panel of 44 primers and probes. This panel was designed based on accumulated findings^14,27^ (including Table 1) to include potential correlates of protection. Across replicate experiments, twelve genes were consistently differentially upregulated in PBLs between vaccine groups (Fig. 4). The patterns of upregulation were similar to that observed with the control of bacterial growth in co-cultures. Genes expressed in cells from the *ΔclpB*- and *ΔclpB-ΔcapB* groups appeared to be upregulated to a similar or greater degree compared to levels observed in PBLs from LVS-vaccinated rats, and gene expression in the PBLs from *ΔclpB-ΔwbtC-* and *ΔclpB*-*ΔfupA-*vaccinated rats was lower than that in cells from LVS-vaccinated rats.

However, the differences between vaccine groups in terms of activities in co-culture assays and gene expression were usually subtle and often not statistically significant. Of note, the results shown in Fig. 2 were combined from four independent experiments, but ten independent experiments were available that evaluated leukocytes from both *ΔclpB*- and LVS-vaccinated rats (Supplementary Fig. 1). When analyzing results from all ten experiments, leukocytes from *ΔclpB*-vaccinated rats provided significantly greater control of intramacrophage bacterial growth compared to those from LVS-vaccinated rats. This suggests the ability to detect subtle differences between levels of intramacrophage growth control in co-culture assays may be limited by sample size.

Real-world applications of correlate measurements are likely to involve small sample sizes and limited assay opportunities. Therefore, we used a multivariate regression analysis (multinomial model; Table 3) to evaluate the power of combined measurements to improve vaccine discrimination. In this approach, the rat co-culture data from bacterial CFU, NO, and the twelve genes analyzed were used to create a profile for each experiment, which was modeled against the vaccine group. The analyses displayed in Table 3 includes the McFadden’s adjusted *R*^2^ values as a measure of model fit, and the Akaike information criterion (AIC), which was calculated as a measure of parsimony for the best models fit by subset size, including each single variable model. Among individual variables, CFU values had the strongest association with the vaccine group, with McFadden = 0.5099. Adding more variables to the model improved model fit, up to a maximum of McFadden = 0.9767 for two models with five variables. After that point, adding more variables did not improve model fit. Moreover, the AIC monotonically increased from AIC = 60 with each additional variable, indicating that the simpler models with five variables provided model fit compared to the models with more variables. Overall, the results for both the McFadden values and AIC suggest that the two best sets of five variables have a robust and parsimonious relationship with the vaccine group.

**Table 3:** Assessment of model fit (McFadden’s Adjusted R^2^) and parsimony (AIC) for the association between different subsets of variables and vaccine group.

Number of variables	Variables	McFadden’s adjusted *R*^2^	AIC
1	CFU	0.5099	60.15
	NO	0.3797	71.35
	Tbet	0.2549	82.08
	CxCr6	0.2012	86.7
	Icos	0.155	90.68
	IL-18bp	0.1133	94.26
	LTA	0.0819	96.96
	CCL5	0.0583	98.99
	IL-21	0.0507	99.65
	Nos2	0.0421	100.38
	IFNγ	0.0295	101.47
	CxCl9	0.0218	102.13
	IL-12rβ2	0.0037	103.69
	FasLg	−0.0141	105.22
2	CFU, CxCr6	0.6998	53.81
	CxCr6, NO	0.5719	64.82
	CFU, NO	0.5281	68.58
	CCL5, IL-21	0.3076	87.55
3	CFU, CxCr6, LTA	0.8831	48.05
	CFU, CxCr6, Nos2	0.7984	55.34
	CFU, CCL5, IL-21	0.7394	60.41
	CFU, CxCr6, IL-21	0.7234	61.79
4	CFU, CxCr6, LTA, IL-12rβ2	0.9466	52.59
	CFU, CxCr6, LTA, IL-18bp	0.8866	57.75
	CFU, CxCr6, IFNγ, IL-21	0.7699	67.79
	CFU, CxCr6, IL-21, NO	0.748	69.68
5	CFU, CxCl9, IL-12rβ2, IL-18bp, LTA	0.9767	60
	CFU, CxCl9, CxCr6, IL-12rβ2, IL-21	0.9767	60
	CFU, CCL5, CxCl9, CxCr6, IL-21	0.9438	62.83
	CFU, CxCl9, CxCr6, IL-21, Tbet	0.9249	64.46
6	CFU, CCL5, CxCl9, CxCr6, IL-21, Tbet	0.9767	70
	CFU, CxCl9, CxCr6, IL-12rβ2, IL-21, Tbet	0.9767	70
	CFU, CxCl9, CxCr6, IL-21, LTA, Tbet	0.9766	70.01
	CFU, CxCl9, CxCr6, FasL, IL-12rβ2, IL-21	0.9353	73.56
7	CFU, CxCl9, CxCr6, FasL, IFNγ, IL-12rβ2, IL-21	0.9767	80
	CFU, CxCl9, CxCr6, FasL, IL-12rβ2, IL-21, Tbet	0.9767	80
	CFU, CxCl9, CxCr6, FasL, IL-12rβ2, IL-21, NO	0.9767	80
	CFU, CxCl9, CxCr6, FasL, IL-12rβ2, IL-21, Nos2	0.922	84.71
8	CFU, CxCl9, CxCr6, FasL, IFNγ, IL-12rβ2, IL-21, Tbet	0.9767	90
9	CFU, CxCl9, CxCr6, FasLg, IFNγ, IL-12rβ2, IL-21, Nos2, Tbet	0.9767	100
10	CFU, CCL5, CxCl9, CxCr6, FasL, IFNγ, IL-12rβ2, IL-21, Nos2, Tbet	0.9767	110
11	CFU, CCL5, CxCl9, CxCr6, FasL, IFNγ, IL-12rβ2, IL-18bp, IL-21, Nos2, Tbet	0.9767	120
12	CFU, CCL5, CxCl9, CxCr6, FasL, IFNγ, IL-12rβ2, IL-21, LTA, Nos2, Tbet, NO	0.9767	130
13	CFU, CCL5, CxCl9, CxCr6, FasL, IFNγ, IL-12rβ2, IL-18bp, IL-21, LTA, Nos2, Tbet, NO	0.9767	140
14	CFU, CCL5, CxCl9, CxCr6, FasL, Icos, IFNγ, IL-12rβ2, IL-18bp, IL-21, LTA, Nos2, Tbet, NO	0.9767	150

Best subsets regression was used to identify models according to McFadden’s adjusted *R*^2^ and Akaike’s Information Criterion (AIC). Values were calculated for each individual variable, for the best four subsets using 2–7 variables; for the best subset using 7–13 variables; or for all variables. High McFadden’s adjusted *R*^2^ values, ranging between 0 and 1, indicate a stronger association with vaccine group. Low AIC values indicate parsimony, in which models with fewer variables provide model fit comparable to models with more variables.

### Serum antibody responses and in vivo protection against aerosol challenge with Type A *F. tularensis* in Fischer 344 rats vaccinated with *ΔclpB*-derived vaccines

**Fig. 5:**
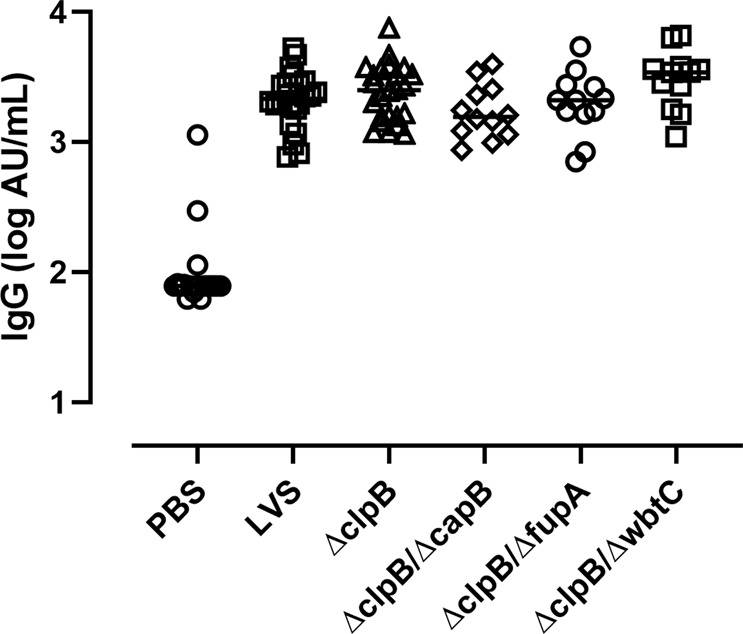
Anti-*Francisella* antibody titers do not show obvious differences across vaccine groups. Fischer rats (*n* = 12–24) were vaccinated as indicated. Thirty-nine days after vaccination, serum samples were collected and analyzed for IgG antibodies using plates coated with heat-killed SchuS4. The results from two independent studies are shown; values indicate the median titer for each sample analyzed from one to four times. All vaccines induced antibodies against SchuS4 (*P* < 0001 vs. PBS), and pair-wise comparisons indicated the only significant difference is between *ΔclpB-Δ*capB and *ΔclpB-ΔwbtC* sera (*P* = 0.03).

To determine the protection afforded by *ΔclpB* and *ΔclpB*-derived vaccines compared to LVS, F344 rats were vaccinated with all live attenuated strains that were studied in vitro. Six weeks later, rats were challenged with fully virulent Type A *F. tularensis* SchuS4 by aerosol inhalation. In an effort to detect differences between vaccine candidates, we used two challenge doses approximately 5-fold apart in each study. Because of limitations on aerosol challenge capacity, we staggered the vaccines administered across independent experiments and included LVS in all experiments as an internal comparator. The averages of presented doses from seven runs were 10,746 ± 4117 CFU/rat (low dose) and 51,668 ± 11,604 CFU/rat (high dose), whereas the averages of lung depositions were 4336 ± 2769 CFU/rat and 9612 ± 6425 CFU/rat, respectively. The LD_50_ for female Fischer 344 rats was estimated to be ~2 CFU/rat, based on lung depositions. We obtained sera from individual animals before vaccination and 3 days prior to challenge, and we then determined relative quantities of IgG anti-*Ft* serum antibodies. *Ft*-specific serum antibodies were readily detectable in all vaccinated rats, and no obvious differences in mean levels were apparent between groups (Fig. 5). Further, although numbers were small, there were no apparent differences in levels between those animals that survived challenge compared to those that did not.

**Fig. 6:**
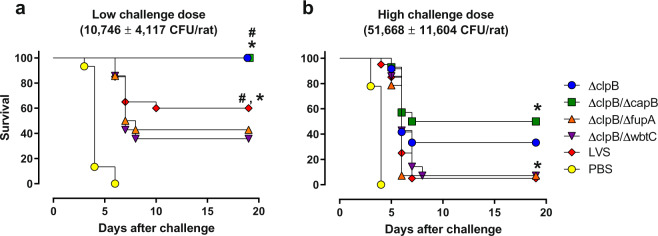
Survival outcomes after aerosol challenge with SchuS4 of vaccinated rats reflect in vitro activities of leukocytes. Fischer 344 rats were vaccinated as indicated. Six weeks after vaccination, rats were challenged by aerosol either with a high (**b**) or low (**a**) dose of SchuS4. Survival was monitored for 28 days. The experiment was repeated three times with various combinations of the vaccines for a total of 8–20 animals per vaccine-challenge dose group. For each challenge dose, bacterial lung deposition was monitored and was comparable across the three experiments. The figure depicts the time to death and survival of results combined from all three experiments. # and * indicate significant differences between survival outcomes for *ΔclpB*- and LVS-vaccinated rats, and between survival outcomes for *ΔclpB-ΔcapB*- and LVS-vaccinated rats, respectively, calculated by Kaplan–Meier and log-rank (Mantel–Cox) analysis.

In challenge studies, the body weight of all animals declined within 3–8 days after the challenge. Thereafter, animals either succumbed or survived and regained weight (Supplementary Fig. 2). All rats treated with PBS died 3–6 days after the challenge (Fig. 6). As expected, more animals in all vaccinated groups survived when given a lower challenge dose (Fig. 6a) compared to those challenged with a higher dose (Fig. 6b). Where differences could be detected, a consistent pattern emerged: *ΔclpB-ΔcapB* and *ΔclpB* (single mutant) provided the strongest levels of protection, which were similar to each other. LVS, *ΔclpB-ΔwbtC*, and *ΔclpB*-*ΔfupA* provided similar levels of protection that were lower than those provided by *ΔclpB-ΔcapB* and *ΔclpB*. To evaluate correlations between in vitro data and protection, we used the data obtained from PBL samples, described in Figs. 2 and 4, and analyzed these in relationship to percent survival. When the low challenge dose data were used, CFU, NO, and relative expression of a few genes were significantly correlated with survival; relative expression of only three genes was significant when the data were evaluated in relationship to the high challenge dose survival (Supplementary Table 1). Thus, with the exception of *ΔclpB*-*ΔfupA*, the hierarchy of activities seen when using cells from vaccinated rats to quantitate intramacrophage growth control in vitro (Figs. 2 and 3), and relative gene expression of the most promising genetic correlates (Fig. 4 and Table 3), was the same as the relative efficacy observed in vivo against the aerosol challenge of vaccinated rats. The dose-dependent correlations with in vivo protection further confirm the value of these measurements, while also illustrating the limitations when measurements from individual parameters are used.

## Discussion

Protection against infection with most intracellular pathogens, including *Francisella*, is highly dependent on T cell-mediated immune responses. Accordingly, to evaluate mechanisms of protection and to develop correlates, we have focused on applying an in vitro co-culture system that measures the ability of a primed T-cell population to control the growth of bacteria within cells, as a functional correlate of vaccine-induced protection. This approach further allows informative measurements of mediators and relative gene expression. Here, we had a unique opportunity to evaluate vaccine candidates in vitro before their in vivo protective capacities were understood, and thus to design in vivo studies to directly test in vitro predictions. Strikingly, the patterns detected in vitro were seen when vaccinated rats were challenged in vivo by aerosol with fully virulent Type A *Ft*. Thus, this strategy provides a valid means to compare vaccines for advanced development. In particular, the in vitro data demonstrated that *ΔclpB* and *ΔclpB*-*ΔcapB* may be better vaccine candidates than LVS, while *ΔclpB-ΔwbtC*- and *ΔclpB*-*ΔfupA* are not. The in vivo data in the rat model confirmed this pattern, consistent with previous data either in mice^33,37^ or rats^34^. Similar studies comparing vaccines against tuberculosis in mice^36,38^ with encouraging results support the applicability of this strategy to evaluating vaccines against intracellular pathogens generally.

Developing effective vaccines against intracellular pathogens has been notoriously difficult, and correspondingly few have been advanced and licensed. The two most obvious examples, BCG for tuberculosis and Ty21a for typhoid fever, are both live attenuated bacterial strains that were generated decades ago and have substantial limitations. In addition to pathogens associated with biowarfare such as *Francisella*, many pathogens of public health concern have an intracellular lifestyle. These include bacteria such as *Chlamydia*, *Brucella*, and *Coxiella*; parasites such as *Leishmania*; and fungi such as *Cryptococcus*. Because vaccine trials involve thousands of people and logistically difficult, other approaches to evaluate and compare new vaccine candidates prior to expanding investment are sorely needed. Such options include applying correlates of protection, particularly during preclinical studies. However, despite many studies, no correlates of vaccine-induced protection against intracellular pathogens have been established in humans to date. Indeed, earlier optimism about deriving correlations between vaccine outcomes and T cell-based assays such as measuring IFN-γ, intracellular cytokine staining, or blood signatures has not yet resulted in meaningful progress^23,39–41^. Correlate analyses are reportedly underway following demonstration of some efficacy in two recent tuberculosis vaccine trials^42,43^ and in NHP vaccine studies^44^, but success remains uncertain. The approach illustrated here to identify functional and molecular vaccine-induced correlates of protection against *F. tularensis* and *M. tuberculosis* continues to lead to promising results^14,25,36,38^.

Our previous studies of LVS and LVS-related vaccines in mouse and rat models to derive correlates of protection were potentially limited by the fact that LVS was derived from Type B *Ft*. Notably, in human studies LVS appeared to provide only partial protection against challenge with Type A *Ft*^7,8^. This has led to interest in developing a vaccine derived from Type A *Ft* strains^29^. Therefore, in this study, we initially explored whether the in vitro co-culture method was suitable for analyzing T cell functions of leukocytes from animals primed with a Type A *Ft*-derived vaccine, namely *ΔclpB*. In BALB/c mice, *ΔclpB* appeared to protect against aerosol challenge with SchuS4 better than LVS^32,45^. Related studies demonstrated that *ΔclpB*-immune mouse splenocytes controlled bacterial growth better than LVS-immune splenocytes when virulent *Ft* SchuS4 was used to infect macrophages^46^. Here, we found the same pattern: when Type B-derived LVS was used as the infecting (and thus re-stimulating) agent, co-cultures with *ΔclpB*-immune PBLs and splenocytes trended toward better control of bacterial replication, more IFN-γ secretion, and more NO production than those with LVS-immune splenocytes (Fig. 1).

Analyses of relative gene expression in recovered mouse lymphocytes further showed that 18 genes, including IFN-γ, NOS2, LTA, FasL, and CCL5, were consistently upregulated in PBLs from *ΔclpB*-vaccinated mice compared to those from LVS-vaccinated mice; other factors, including IL-21, GzmB, and T-bet, were expressed similarly in *ΔclpB*- and LVS-immune cells (Table 1). Collectively, these data demonstrated that the co-culture approach is appropriate and applicable across bacterial sub-strains. In other studies, using a similar approach, some mechanisms of intramacrophage growth control differed when mouse macrophages were infected in vitro with LVS compared to those infected with fully virulent *F. tularensis* SchuS4^47,48^. Nonetheless, studies with co-cultures utilizing SchuS4-infected rat macrophages resulted in similar results as seen here^34^. Therefore, for the purposes of in vitro co-culture studies between infected macrophages and immune lymphocytes, LVS, which can be used in a BSL-2 laboratory, appears to be a useful surrogate for virulent Type A *Ft*, which requires BSL-3 containment. Moreover, the data not only suggest induction of stronger immune responses by *ΔclpB* immunization, but also suggests potential refinements of the working gene panel.

Of note, survival studies performed with mice vaccinated with *ΔclpB* or LVS or with all four *ΔclpB*-derived vaccines did not discriminate between vaccines, highlighting the limitation of the mouse model. We, therefore, turned to the F344 rat model to further evaluate *ΔclpB* and to screen additional *ΔclpB*-derived vaccines. Although we observed some experiment-to-experiment variability, the control of in vitro LVS intramacrophage replication, the production of NO, and the secretion of IFN-γ from cells obtained from *ΔclpB*- and *ΔclpB-ΔcapB*-vaccinated rats were consistently comparable to each other and often trended better than those from LVS-vaccinated rats (Figs. 2 and 3 and Supplementary Fig. 1). In contrast, leukocytes from *ΔclpB-ΔwbtC*- and particularly *ΔclpB*-*ΔfupA*-vaccinated rats exhibited less growth control activity than those from LVS-vaccinated rats.

We found a similar hierarchy of relative gene expression when studying a working panel of correlate genes (Fig. 4), most of which were selected previously to reflect the relative efficacy of LVS-related vaccines in rats^14,27^. Our previous studies of a panel of LVS-related vaccines in rats demonstrated the importance of combining measured parameters, including bacterial replication and relative gene expression^14,35^. Here, any single measurement was modestly associated with vaccine group. In particular, only the relative gene expression values obtained using cells from *ΔclpB*-vaccinated animals of CCL5, CXCR6, FASL, and IL-18bp; the *ΔclpB-ΔcapB* value of CCL5; the *ΔclpB-ΔwbtC* value of NOS2; and the *ΔclpB*-*ΔfupA* values of IL-21 and LTA were significantly different than those of LVS (*P* < 0.05). In light of the subsequent in vivo protection data, the mediators that distinguished survival outcomes merit further study for their functional and predictive significance.

Combining bacterial replication data (Fig. 2a), NO production (Fig. 2c) and relative gene expression of 5–12 genes (Fig. 4) in multivariate models (multivariate regression analysis) greatly increased the strength of the association with the vaccine group (Table 3). Due to the relatively small sample sizes, using cross-validation to identify the variables, (as previously done^14^) would be inappropriate. Instead, we used best subsets regression to identify subsets of variables which had a robust relationship with the vaccine group. As seen earlier^14,35^, CFU values provided the strongest association with the vaccine group, followed by NO. The relationship with NO is consistent with previous findings^46^. However, when CFU and NO were evaluated in combination, we observed only a marginal improvement in model fit compared to CFU alone. This was likely due to multicollinearity, in which CFU and NO may have high overlap in their relationships with the vaccine group; thus, only marginal benefit resulted from including both. Moreover, the accuracy in modeling the vaccine group improved with the addition of up to five variables, which resulted in the identification of subsets with the highest McFadden values. Additionally, the increase in AIC for five variables and above indicates that five variables provided fit comparable to models with 6–14 variables. We did not fit models with CFU (or any other individual variable) as the response variable. Therefore, we cannot comment on any potential relationship between CFU and groups of genes. However, we found that CFU along with CXCL9, IL-12rb2, IL-18bp, and LTA, or other combinations of five or more genes, had a strong relationship with vaccine types.

Most importantly, the pattern of relative vaccine efficacy determined using in vitro co-cultures, including relative expression of selected gene correlate candidates, was largely reflected by survival outcomes when rats were vaccinated and challenged by aerosol with fully virulent Type A *Ft* (Fig. 6). In both mouse and rat studies, measurements of in vitro functions indicated that *ΔclpB* and *ΔclpB-ΔcapB* may represent better vaccines than LVS, while *ΔclpB-ΔwbtC* and *ΔclpB*-*ΔfupA* may not. Consistent with our assessments of *ΔclpB-ΔwbtC*, previous in vitro co-culture and in vivo challenge studies also showed that *ΔclpB-ΔwbtC* vaccination engenders suboptimal T-cell function and protection compared to LVS^34^. Indeed, in vitro co-culture studies detected subtle differences between vaccines that may not be readily demonstrated by in vivo challenge, even when using two doses to seek breakthrough challenge dose points.

The exception to the overall patterns observed was seen with the double mutant *ΔclpB*-*ΔfupA*, with a discrepancy between in vitro co-culture outcomes and in vivo survival. Although rodents may not represent the best models to evaluate the contributions of vaccine-induced antibody responses against *Ft*^49^, this discrepancy could not be readily explained by additional circulating anti-*Ft* antibodies in *ΔclpB*-*ΔfupA*-vaccinated rats (Fig. 5). Differences may be due to levels of antibodies in lungs, which may play a role, or to the nature of the *ΔfupA* mutant itself. Deletion of *fupA*, which regulates iron uptake in *Ft*^50,51^, results in increased secretion of outer membrane vesicles^52^. These altered phenotypes may stimulate immune responses that are not measured by in vitro co-culture assays and/or different in vitro effectors mechanisms when an attenuated bacterial strain is used to infect macrophages compared to a fully virulent *Francisella* strain^47,48^. Future studies will continue to further explore the potential limitations of the co-culture approach.

The survival patterns now provide a rational approach to further refine the working panel of genetic correlates (Fig. 4), currently the most useful across our series of studies, and to focus on genes reflecting survival patterns. To that end, we conducted exploratory analyses in mice of relative expression of genes that were not obviously related to immune functions. Indeed, we found that 30 genes were expressed in the pattern *ΔclpB* ≥ LVS > LVS-R > PBS, of which 19 were upregulated more in *ΔclpB*-immune cells than in LVS-immune cells (Table 2). Conversely, we have not found genes that were solely upregulated in LVS-immune cells but not in *ΔclpB*-immune cells. This suggests the activation of similar pathways and supports the conclusion that vaccination with *ΔclpB* induces a broader and stronger set of immune responses than vaccination with LVS, even when re-stimulated by LVS in macrophages. Future studies will continue to better evaluate the pathways involved during ex vivo re-stimulation of primed lymphocytes and, ultimately, to refine and model all available data to provide the strongest predictions. Moreover, the data shown here confirm that CFU measurements provide not only the strongest association with vaccine groups (Table 3), but also correlate with survival (Supplementary Table 1). Although a few individual genes showed correlations with survival after low challenge dose, the combination of 5–7 measurements, including CFU, substantially improved associations with vaccine group^14^ (Table 3). In the present study, the combinatorial analyses were designed to discriminate between all five vaccine groups and one naive group. Now that in vivo challenge studies have identified a hierarchy of protection among these groups of vaccines, future analyses could be directed to seeking combinations that discriminate between the three groups identified by in vivo challenge studies (Fig. 6). However, meaningful multivariate analyses with sufficient power to examine correlations between groups of parameters and survival would require survival studies using substantially more animals, which is problematic for humane reasons.

Finally, 13 of the upregulated genes were differentially expressed during both anti-*Ft* (Table 2) and anti-*M. tuberculosis*^36^ responses. This finding suggests some similar immune mechanisms control both pathogens and further emphasizes the potential utility of this approach for other intracellular bacteria. Importantly, this raises the possibility of identifying universal biomarkers to screen vaccines against intracellular bacteria generally.

Taken together, the accumulated results strongly support the overall value of the in vitro co-culture strategy for the purposes of evaluating vaccine candidates against intracellular bacteria such as Type A *Ft*. In particular, the in vitro data identified *ΔclpB* and *ΔclpB-ΔcapB* vaccines as better alternatives to LVS. Similar assays have been developed using human monocytes and lymphocytes^53–56^ as well as using PBLs from non-human primates (unpublished data). Thus, the present studies support advancing this approach to studies with non-human primates and people, in order to compare vaccine candidates and to bridge between species.

## Methods

### Experimental animals

Six- to twelve-week-old specific-pathogen-free male C57BL/6J were purchased from Jackson Laboratories (Bar Harbor, ME); 6–11-week-old female Fischer 344 rats were purchased from Envigo (Indianapolis, IN). For studies performed at CBER/FDA, all animals were housed in sterile microisolator cages in a barrier environment, fed autoclaved food and water ad libitum, and routinely tested for common murine pathogens by a diagnostic service provided by the Division of Veterinary Services, CBER. For studies performed at UNMHSC, all animals were housed in individually ventilated cages (Techniplast, Italy) in an ABLS-2 laboratory before the challenge and in a CDC-certified Select Agent ABSL-3 laboratory after the challenge. The animals were fed ad libitum with Teklad irradiated rodent chow #2920x (Envigo) and given chlorine dioxide-treated filtered water. Within an experiment, all animals were age-matched. All experiments were performed under protocols approved by the Animal Care and Use Committee of CBER (Animal Study Protocols #1993-03 and #2015–21) and UNMHSC (Animal Study Protocol 14-101234-HSC and 19-200938-HSC). These protocols meet the standards for humane animal care and use set by the Guide for the Care and Use of Laboratory Animals and U.S. Public Health Service policy. Infection studies included frequent observations and observed humane endpoints. At the indicated time points or at the end of a study, animals were euthanized with carbon dioxide inhalation in a euthanasia chamber where carbon dioxide was introduced at the rate of at least 20% of the chamber volume per minute or with sodium pentobarbital (Fatal-Plus; Vortech Pharmaceuticals, Dearborn, MI). The health status of the vaccinated and challenged animals was monitored and the stage when the animals did not markedly move, even in response to physical stimulus, and therefore were unable to reach water and food was considered a sign of imminent death. The animals were then sacrificed, and the time of sacrifice recorded as the time of death.

### Bacteria and growth conditions

*F. tularensis* LVS (American Type Culture Collection 29684), SchuS4 *ΔclpB*, and the double mutants SchuS4 *ΔclpB*-*ΔfupA*, SchuS4 *ΔclpB*-*ΔcapB*, and SchuS4 *ΔclpB*-*ΔwbtC* were grown to mid-log phase in modified Mueller-Hinton (MH) broth (Difco Laboratories, Detroit, MI), harvested, and frozen in aliquots in broth alone at −80 °C^57,58^. *F. tularensis* strain SchuS4 (BEI Resources, Manassas, VA, NR-28534) was sub-cultured in Modified Cysteine Partial Hydrolysate (MCPH) broth to produce a sub-master stock and a working stock, which were frozen in aliquots with 20% glycerol at −80 °C. Bacteria were periodically thawed for quality control by quantification of viability on MH agar plates.

### Bacterial immunizations

Mice were immunized by i.d. injection with 1 × 10^4^ colony forming units (CFU) of the vaccine strains, diluted in 0.1 ml phosphate-buffered saline (PBS) (BioWhittaker/Lonza, Walkersville, MD). Alternatively, rats were anaesthetized with isoflurane and immunized by s.c. administration of 5 × 10^6^ to 1 × 10^7^ CFU of the vaccines. Control groups received PBS. Actual doses of administrated vaccine were determined by retrospective plate count.

### Serum collection

Two or three days before vaccination and three days before aerosol challenge, 300 μl of blood was collected from the tail vein of vaccinated rats using 21 G blood collection sets (Terumo Medical Products; Somerset, NJ) into microcentrifuge tubes and centrifuged at 8500 × *g* for 5 min at room temperature. The sera were transferred into fresh microcentrifuge tubes and stored at −20 °C until use. After the serum samples were thawed, they were stored at 4 °C until all analyses were completed.

### Survival studies

Survival studies in mice were performed by i.p. administration of 1 × 10^6^ CFU LVS^16,25,26,35^. Animals were monitored for survival for at least 30 days. Aerosol challenges were performed in naive and vaccinated Fischer 344 rats^59^. Briefly, SchuS4 infections solutions were prepared in brain heart infusion broth (BHIB; Teknova; Hollister, CA) to desired concentrations based on the historical correlation between generator concentration and lung deposition. SchuS4 aerosols were generated using a Collison 3-jet nebulizer (BGI, Inc., Waltham, MA) and presented to rats in a nose-only exposure chamber (In-Tox Products, Inc., Moriarty, NM). Due to the large numbers, the animals in each study were divided and exposed over multiple exposure runs. For each run, an impinger sample was plated to determine exposure concentration. In particular, the presented dose for each exposure run was calculated using the measured impinger concentration from a 20 ml impinger at a rate of 5 L/min for 15 min exposure, and the respiratory minute volume was estimated using the Guyton formula and body weight. The average weight of all study animals in 2 studies were 179 g and 192 g, respectively (Supplementary Fig. 2). Body weights were not recorded in one study but were estimated to be 175 g based on published growth curves. One rat was included in each exposure run and euthanized after all exposure runs for the day were completed. The lungs were homogenized and plated to enumerate viable bacteria deposited in the lungs. The LD_50_ for female F344 rats was estimated to be ~2 CFU/rat, based on lung depositions. Rats were monitored for survival for at least 20 days, and their body weight was recorded.

### Co-culture of bone marrow-derived macrophages with leukocytes

Co-cultures were performed 6–7 weeks after vaccinations. Briefly, 2 × 10^6^ or 1 × 10^6^ ACK-treated cells from bone marrow-derived macrophages of naive mice or rats, respectively, were cultured in DMEM supplemented with 10% heat-inactivated FCS (HyClone, Logan, UT), 10 ng/ml mouse or rat CSF^60^, 0.2 mM L-glutamine, 10 mM HEPES buffer, 1 mM sodium pyruvate, 1 mM sodium bicarbonate and 0.1 mM non-essential amino acids, in 24-well plates. After 7 days of incubation with media changes, confluent macrophages were infected for 2 h with LVS at a multiplicity of infection (MOI) of 1:20 (for mouse co-cultures) or of 1:50 (for rat co-cultures), then washed, treated with 50 µg/ml gentamicin, and washed^60^. Isolated spleens and blood were used to prepare single-cell suspensions of splenocytes and PBLs; erythrocytes were lysed with ammonium chloride (ACK lysing buffer, BioWhittaker/Lonza). Cells were washed with PBS 2% FCS, viability was assessed by the exclusion of trypan blue, cell concentrations were adjusted as required, and 5 × 10^6^ cells/well, pooled from multiple animals were co-cultured with LVS-infected macrophages. After 2 days culture non-adherent cells were recovered, pelleted, and stored in RNA*later* (Ambion, Austin, TX) at −80 °C for further analyses; when required, an aliquot of recovered cells was used for surface staining and flow cytometry. Supernatants were collected after 2 or 3 days of co-culture and stored at −80 °C for further analysis, and adherent LVS-infected macrophages were lysed at the indicated time point for determining LVS intramacrophage replication by plate count.

### Flow cytometry

Single-cell suspensions were prepared from total splenocytes and PBLs, and from cells recovered from co-cultures. Cells were incubated with anti-CD16/CD32 (Fc block, BD Pharmingen, San Diego, CA) and stained with Live/Dead staining kit (Invitrogen, Carlsbad, CA). Cells were then washed in flow cytometry buffer (PBS with 2% FBS) and stained for cell surface markers. Antibody concentrations were previously optimized for use in multi-color staining protocols as required, using appropriate fluorochrome-labeled isotype-matched control antibodies. The following antibodies were used: anti-B220 (clone RA3-6B2), anti-CD19 (clone 1D3), anti-TCRβ (clone H57-597), anti-CD4 (clone RM4-5), anti-CD8β (H35-17.2), anti-NK1.1 (clone PK136), anti-CD11b (clone M1/70), anti-Gr-1 (clone RB6-8C5), and anti-CD11c (cloneHL3). A minimum of ten thousand total events were counted using an analytical LSR II or LSR Fortessa flow cytometer (Becton Dickinson). Data analyses were performed using FlowJo (Tree Star, Inc.) software using a gating strategy as shown in Supplementary Fig. 3.

### Real-time PCR

Cells stored in RNA*later* at −80 °C were used to purify total RNA using RNeasy mini kits (Qiagen, Valencia, CA), according to the manufacturer’s directions. One microgram of RNA was used to synthesize cDNA using the commercially available kit High Capacity RNA-to-cDNA (Applied Biosystems, Carlsbad, CA), following the manufacturer’s instructions. Semi-quantitative real-time PCR amplification was completed with an ABI Prism 7000 sequence detection system (Applied Biosystems, Carlsbad, CA). For screening of relative expression of mouse genes, cDNA was used in two separate sets of semi-quantitative real-time PCR (ViiA 7 sequence detection system, Applied Biosystems). Specifically, two mouse custom arrays were designed using Applied Biosystem primers and probes. The first array contained 92 genes of immunological interest plus three housekeeping genes and one control (Supplementary Table 2); genes were selected based on the outcomes obtained in previous experiments of similar design^14,27^. The second array contained 188 genes, including three housekeeping genes (Supplementary Table 3). To select the latter genes, experiments of a similar design were performed by vaccinating mice with BCG Pasteur; PBLs were then recovered from co-cultures and used for gene expression analyses by microarray, and data were then validated for 188 genes using Real-Time PCR^36^.

For rat gene expression studies, one rat custom array was designed using Applied Biosystem primers and probes, containing 44 genes of immunological interest plus three housekeeping genes and one control (Supplementary Table 4); genes were selected based on the outcomes obtained in the mouse experiments shown here and in previous studies^14,27^. Alternatively, a limited series of independent amplifications for selected genes were performed. Independent primers and probes were purchased from Applied Biosystems (Supplementary Table 5). In all qRT-PCR studies, Gusb, RPS29, and GAPDH were used to normalize data; delta Ct (ΔCt) and the ratio between ΔCt of vaccine samples and control samples (ΔΔCt) were then calculated.

### Assessment of supernatants

Supernatants recovered on days two or three from in vitro co-cultures were assessed for IFN-γ and nitric oxide production. Quantification of IFN-γ was assessed using standard sandwich ELISAs, per the manufacturer’s instructions (BD Pharmingen, San Diego, CA) by comparison to recombinant standard proteins (BD Pharmingen). Estimation of nitric oxide (NO) was performed using the Griess reaction^61^ (Sigma-Aldrich, St Louis, MO) and by comparison to serially diluted NaNO_2_.

### Assessment of serum antibody levels

Anti-*Francisella* rat serum antibody levels were evaluated by a modified ELISA based on methods for studies of mouse antibodies^26,62,63^. Briefly, flat-bottom ELISA plates (Thermo Fischer Scientific, Waltham, MA) were coated with 10^7^ CFU/well of heat-killed SchuS4 in 100 μL of coating buffer (32 mM Na_2_CO_3_, 68 mM NaHCO_3_, pH 9.6) overnight at 4 °C. The plates were washed three times with 200 μL wash solution (PBS with 0.05% (v/v) Tween-20) and blocked with 200 μL of blocking solution (PBS with 10% liquid gelatin; Norland Products; Cranbury, NJ) for 1 h at room temperature. Serum samples were serially diluted in blocking solution, added to the plates, and incubated for 1–2 h at room temperature. A reference SchuS4 antiserum was used to quantitate antibody responses. The reference antiserum was collected and pooled from four female rats 2 weeks after s.c. inoculation with 1.45 × 10^3^ CFU SchuS4. The pooled antiserum was rendered non-infectious by filtration through a 0.22-μm polyethersulfone membrane syringe filter (MilliporeSigma; St. Louis, MO) and verified as sterile by plating 10% of the sample volume onto chocolate agar plates (Hardy Diagnostics; Santa Maria, CA), then stored in aliquots at −20 °C. The pooled antiserum was assigned a titer of 1000 Arbitrary Unit (AU)/mL. Serial dilutions from 0.15 to 2.5 AU/mL were included on every plate as reference standards. Bound antibodies were detected using horseradish peroxidase-conjugated goat anti-rat IgG (Southern BioTech; Birmingham, Al) and 3,3′,5,5′-tetramethylbenzidine as substrate (EMD Millipore Calbiochem; Billerica, MA). The reaction was stopped using 100 µL of 0.5 N H_2_SO_4_, and plates were then read at 450 nm in a µQuant microplate reader running Gen5 software version 2.01.14 (BioTek; Winooski, VT). A 4-parameter logistic curve was fitted to the reference standards on each plate and used to interpolate the titer of *Ft* SCHU S4-specific IgG antibodies. Each serum sample was analyzed up to four times and the median value was reported.

### Statistical analyses

Microsoft Excel was used to evaluate differences in bacterial growth, IFN-γ production, and gene expression. CFU data were log_10_ transformed and cytokine concentrations were measured using a log scale; thus, a normal distribution was assumed. Significant differences were evaluated using a two-tailed Student’s *t* test, with a *P* value of <0.05 indicating significance. Corrections for multiple comparisons were performed using the Bonferroni method. To determine which correlates of protection had the strongest association with the vaccine group, we initially selected measurements of CFU, nitric oxide, and relative expression of twelve genes as variables or correlates and used data to fit a multinomial regression model, as illustrated in Table 3. Specifically, we used best subsets regression to optimize model fit for each subset size ranging from 1 to 14 variables and assessed models according to McFadden’s Adjusted *R*^2^. To differentiate between models with similar McFadden values, we calculated Akaike’s Information Criterion (AIC) as a measure of parsimony, which indicated that simpler models provide comparable model fit relative to models with more variables. Comparisons of survival curves between different groups of rats were statistically evaluated by Kaplan-Meier and Log-rank (Mantel-Cox) analysis (GraphPad Software, Version 9.1.1, San Diego, CA, USA). Comparisons of anti-Ft IgG antibody titers were evaluated by one-way ANOVA with Tukey’s multiple comparisons test (GraphPad Software). Non-parametric Spearman correlations were evaluated by plotting the CFU, NO, and IFN-γ values derived from PBLs (Fig. 2) and the gene expression data (Fig. 4) against percent survival after low and high challenge doses (Fig. 6) of the corresponding vaccine group using GraphPad Software.

### Reporting summary

Further information on research design is available in the Nature Research Reporting Summary linked to this article.

## Supplementary information

Supplementary Material Merged

REPORTING SUMMARY

## Data Availability

Data generated or analyzed during this study are included in this published article and its supplementary information files; data are available from the corresponding author on request.
