# Supplementary material for: Working correlates of protection predict SchuS4-derived-vaccine candidates with improved efficacy against an intracellular bacterium, Francisella tularensis
Source: NPJ Vaccines. 2022 Aug 17;7:95. doi: 10.1038/s41541-022-00506-9 (PMC9385090; doi:10.1038/s41541-022-00506-9)
Supplement: Supplementary file 1 — Supplementary Material Merged [file 41541_2022_506_MOESM1_ESM.pdf]

Supplementary Figure 1

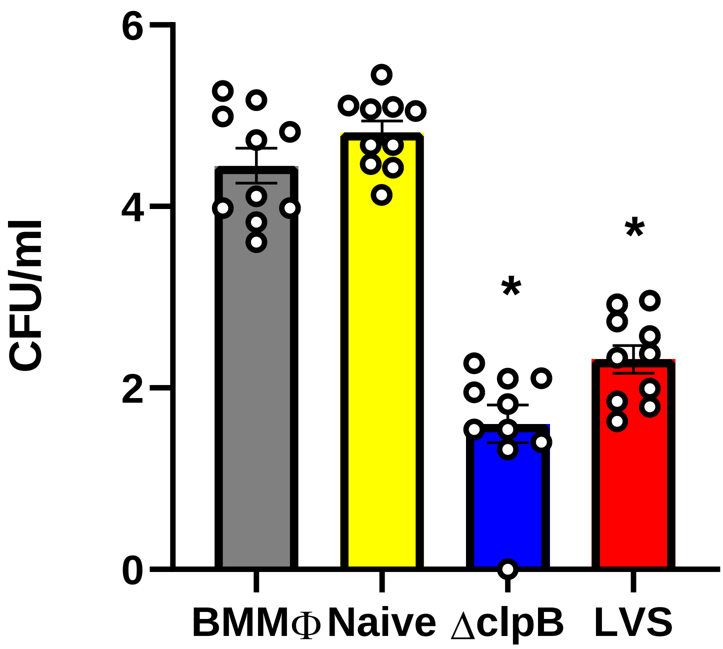

PBLs from *ΔclpB*-vaccinated rats exhibited better control of bacterial replication than those from LVS-vaccinated rats. BMMΦ from Fischer 344 rats were infected with LVS and co-cultured with PBLs obtained from naïve or *ΔclpB*- and LVS-vaccinated rats. After two days of co-culture, BMMΦ were lysed to evaluate the recovery of intracellular bacteria. Values shown are the average from ten independent experiments of similar design. Error bars indicate standard error of the mean (s.e.m.). Asterisks indicate significant differences between the two vaccine groups ($p = 0.0226$), calculated by 2-way ANOVA.

Supplementary Table 1. Spearman correlation between values derived from PBL samples and percent survival.

	Low challenge dose		High challenge dose	
	Spearman r	P value	Spearman r	P value
CFU	-0.928	0.022	-0.812	0.061
NO	0.928	0.022	0.812	0.061
IFN protein	0.754	0.106	0.406	0.444
CXCL9	0.986	0.006	0.754	0.094
IL-18bp	0.986	0.006	0.754	0.094
CXCR6	0.926	0.022	0.754	0.094
ICOS	0.899	0.028	0.522	0.300
T-BET	0.812	0.072	0.899	0.028
IL-12r 2	0.812	0.072	0.841	0.044
IFN	0.754	0.106	0.348	0.506
CCL5	0.754	0.106	0.928	0.017
IL-21	0.754	0.106	0.281	0.590
LTA	0.754	0.106	0.348	0.506
FASL	0.551	0.272	0.783	0.072
NOS2	0.522	0.300	0.116	0.839

Non-parametric Spearman correlation was calculated as described in Material and Methods. Two-tailed P value <0.05 indicates significance. The negative value obtained with CFU is indicative of the inverse correlation between reduction of bacteria proliferation and survival.

Supplementary Figure 2

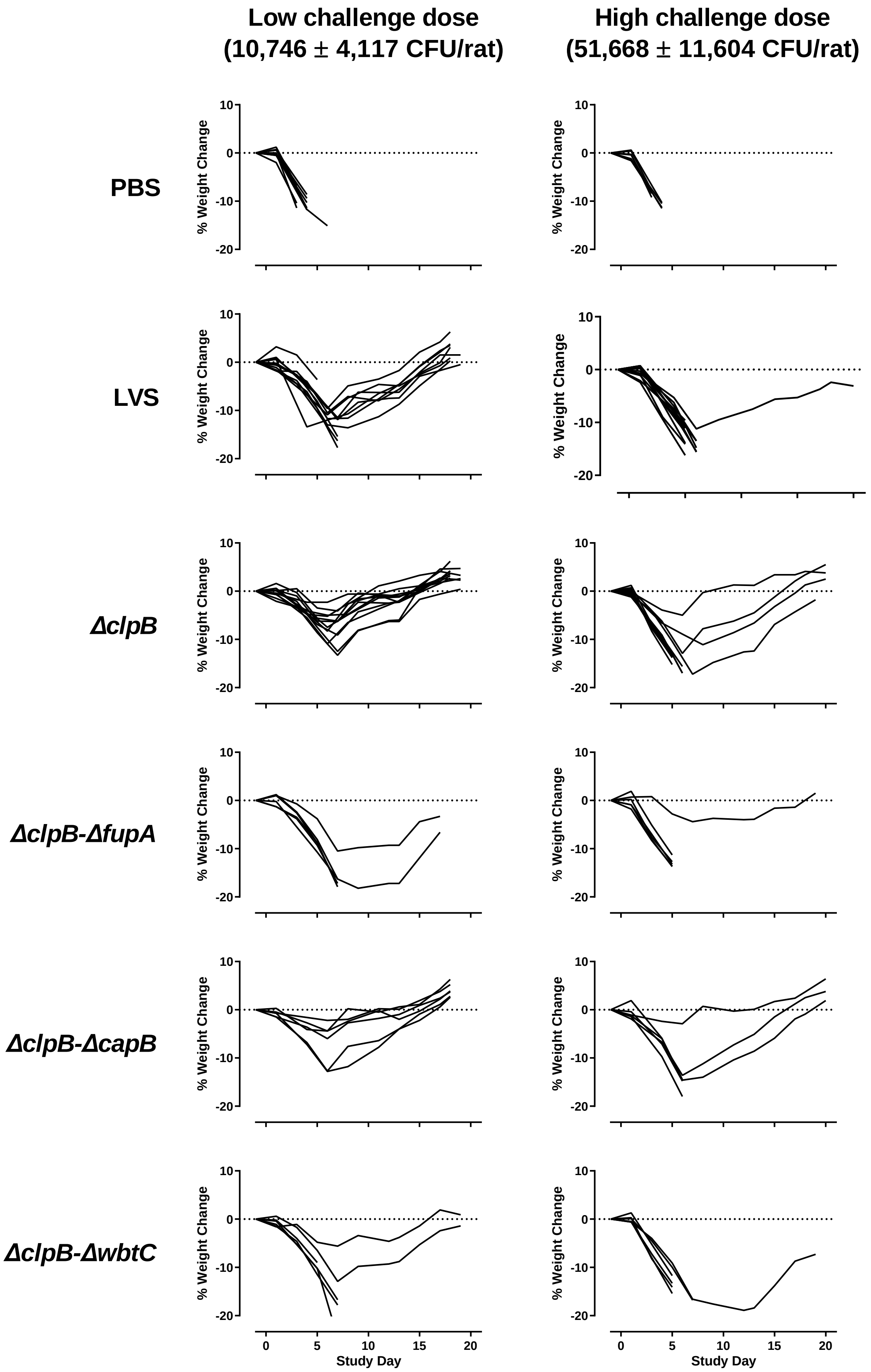

Body weight of aerosol challenged rats reflects survival outcomes. Fischer 344 rats were vaccinated as indicated. Six weeks after vaccination, rats were challenged by aerosol either with a high or low dose of SchuS4. Body weight was monitored for 28 days. The experiment was repeated two times with various combinations of the vaccines for a total of 6 – 12 animals per vaccine-challenge dose group. The figure depicts result combined from two experiments.

Supplementary Figure 3

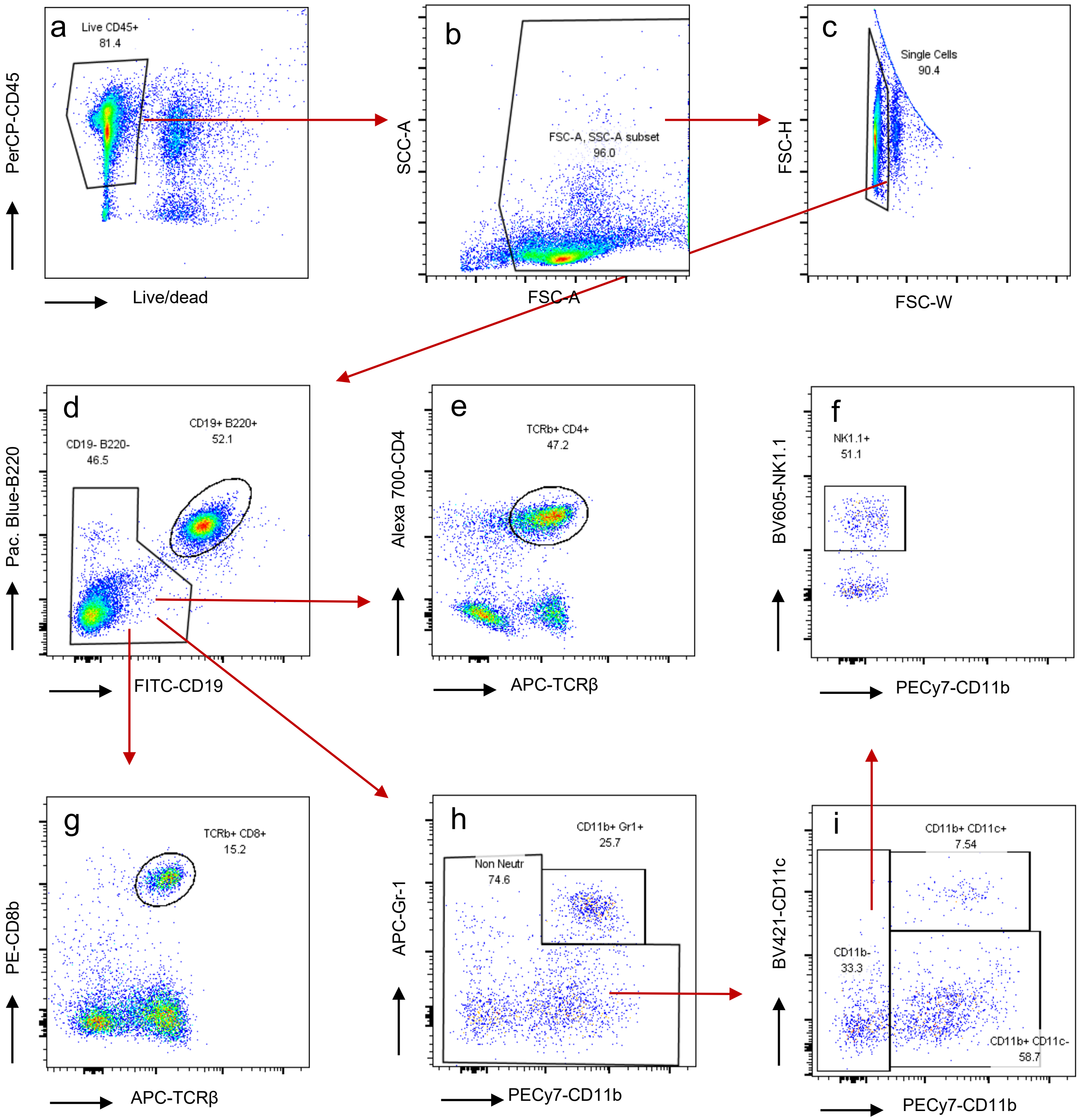

Example of gating strategy. After removing dead cell (a), fragments (b) and aggregates (c), CD45^{+} cells were gated with CD19 and B220 to quantify B cells (d). To evaluate T cells (e and g) and neutrophils (h), non-B cells were gated using TCR , CD4, CD8, and CD11b and Gr1, respectively. The remaining cells were further gated with CD11c and NK1.1 to identify dendritic cells and macrophages (i), and natural killers (f).

Supplementary Table 2: Primers for custom mouse PCR array #1

Assay ID	Gene Symbol(s)	Gene Name(s)	Gene Alias(es)	GenBank mRNA(s)	Amplicon Length
Mm00432051_m1	Bax	mCG23178 Celera Annotation;BCL2-associated X protein	-	BC053380.1;L22472.1;BC018228.1;AK149994.1;AK159233.1;AK013298.1;AY095934.1	84
Mm00477631_m1	Bcl2	mCG128637 Celera Annotation;B cell leukemia/lymphoma 2	AW986256;Bcl-2;C430015F12Rik;D630044D05Rik;D830018M01Rik	AK049473.1;BC095964.1	85
Mm00437797_m1	Bmp15	mCG16375 Celera Annotation;bone morphogenetic protein 15	AU015375;AU018861;AU021453;Bmp-15;C86824;C87336;GDF-9B	AK135757.1;AK139936.1;AJ132406.1;AF082348.1;BC055363.1;AK139773.1;AK135882.1;AK139847.1;AK136129.1;AK028735.1;AK136127.1	64
Mm00437783_m1	Bcl2l1	BCL2-like 1;mCG50852 Celera Annotation	Bcl(X)L;Bcl-XL;Bcl2l;BclX;bcl-x;bcl2-L-1	BC089016.1;BC089017.1;X83574.1;L35049.1;AK172250.1;U10101.1;AK146461.1;U51278.1;AK150028.1	65
Mm01232779_m1	C3	complement component 3;mCG140909 Celera Annotation	AI255234;ASP;HSE-MSF;Plp	K02782.1;DQ408205.1;EU868829.1;BC043338.1;BC029976.1;HM856604.1	88
Mm00443111_m1	Ccl4	mCG11627 Celera Annotation;chemokine (C-C motif) ligand 4	AT744.1;Act-2;MIP-1B;Mip1b;Scya4	AK172472.1;M23503.1;M35590.1;AF128218.1;AK156948.1;BC119259.1;AF128219.1;BC119257.1;BY163868.1	70
Mm01302427_m1	Ccl5	mCG11684 Celera Annotation;chemokine (C-C motif) ligand 5	MuRantes;RANTES;SISd;Scya5;TCP228	AK003101.1;AK158074.1;M77747.1;BC033508.1;AF065944.1;AF065947.1;AF065945.1;AY722103.1;AF065946.1;AF128187.1;S37648.1;CT010315.1	103
Mm00443113_m1	Ccl7	chemokine (C-C motif) ligand 7;mCG52384 Celera Annotation	MCP-3;Scya7;fic;marc;mcp3	AF128194.1;AF128193.1;S50588.1;BC061126.1;S71251.1;AK078827.1;Z12297.1;L04694.1;BY163435.1	122
Mm00839967_g1	Ccl19	chemokine (C-C motif) ligand 19	CKb11;ELC;MIP3B;Scya19;exodus-3	BC051472.1;AF059208.2;AK144337.1	79
Mm00445039_m1	Ccl28	chemokine (C-C motif) ligand 28;mCG114447 Celera Annotation	CCK1;MEC;Scya28	BC055864.1;AF220238.1;BC040510.1	89
Hs99999901_s1	18s rRNA	-	-	-	0
Mm00438260_s1	Ccr1	chemokine (C-C motif) receptor 1;mCG16377 Celera Annotation	Cmkbr1;Mip-1a-R	U29678.1;AK036597.1;AK076275.1;AK036690.1;CT010178.1;BC011092.1;AK031109.1	66
Mm00438270_m1	Ccr2	chemokine (C-C motif) receptor 2;mCG16379 Celera Annotation	Cc-ckr-2;Ccr2a;Ccr2b;Ckr2;Ckr2a;Ckr2b;Cmkbr2;mJe-r	BC138803.1;AK046579.1;AK138599.1;BC138804.1;U56819.1;U51717.1	100
Mm00515543_s1	Ccr3	chemokine (C-C motif) receptor 3;mCG16380 Celera Annotation	CC-CKR3;CKR3;Cmkbr1l2;Cmkbr3	BC108967.2;AK153518.1;AK041106.1;AK089895.1;BC108968.1;AK089875.1	100
Mm01963217_u1	Ccr4	mCG7123 Celera Annotation;chemokine (C-C motif) receptor 4	C-C CKR-4;CHEMR1;Cmkbr4;LESTR;Sdf1r	BC119171.1;AK042013.1;X90862.1;BC117041.1	72
Mm01963251_s1	Ccr5	mCG16378 Celera Annotation;chemokine (C-C motif) receptor 5	AM4-7;CD195;Cmkbr5	AK155632.1;BG091923.1;AK154595.1;AK171391.1;D83648.1;AK155628.1	153
Mm02528165_s1	Ccr9	chemokine (C-C motif) receptor 9;mCG15844 Celera Annotation	A130091K22Rik;Cmkbr10;GPR-9-6	AK019478.1;AJ131357.2;AK050615.1;AK153975.1;AK162762.1	67
Mm00437153_m1	Tnfsf8	mCG20076 Celera Annotation;tumor necrosis factor (ligand) superfamily, member 8	CD153;CD30LG;Cd30l	AK030916.1;BC117093.1;BC117097.1;L09754.1;AK157270.1	85
Mm00441891_m1	Cd40	mCG17530 Celera Annotation;CD40 antigen	AI326936;Bp50;GP39;HIGM1;IGM;IMD3;T-BAM;TRAP;Tnfrsf5;p50	AJ401389.1;AJ401388.1;AK156644.1;AJ401390.1;AK162305.1;AJ401387.1;AK150959.1;AK089861.1;AK161978.1;AK151458.1;BY743050.1;AK152716.1;AK152756.1;BY259264.1;M83312.1;AK152942.1	68

Mm00441911_m1	Cd40lg	CD40 ligand;mCG17341 Celera Annotation	CD154;CD40-L;Cd40l;HIGM1;IGM;IMD3;Ly-62;Ly62;T-BAM;TRAP;Tnfsf5;gp39	X65453.2;EU274582.1;BC119225.1	120
Mm00839636_g1	Cd68	CD68 antigen	Lamp4;Scard1;gp110	X68273.1;AK002264.1;AW123877.1;AK170443.1;BC021637.1	86
Mm00441914_m1	Cd70	mCG5599 Celera Annotation;CD70 antigen	CD27LG;Cd27l;Tnfsf7	BC125407.1;BC125409.1;U78091.1;Y13636.1	66
Mm00711660_m1	Cd80	mCG129101 Celera Annotation;CD80 antigen	B71;Cd28l;Ly-53;Ly53;MIC17;TSA1	AF065895.1;AF065896.1;AF065893.1;AF065894.1;BC145843.1;X60958.1;AK154329.1;BC131959.1;AJ278965.1;AY278186.1	117
Mm00444543_m1	Cd86	mCG130169 Celera Annotation;CD86 antigen	B7;B7-2;B7.2;B70;CLS1;Cd28l2;ETC-1;Ly-58;Ly58;MB7;MB7-2;TS/A-2	L25606.1;AF065899.1;AF065900.1;AF065897.1;S70108.1;AF065898.1;BC013807.1;AK079513.1;AK170054.1;AK172548.1	87
Mm00470315_m1	Cmtm3	mCG21549 Celera Annotation;CKLF-like MARVEL transmembrane domain containing 3	9430096L06Rik;AI413895;BNAS2;Cklfsf3	AK003882.1;AK079165.1;AY241870.1;AK172091.1;BC145817.1;BC145819.1;BC003230.1	67
Mm00463816_m1	Cmtm4	mCG21554 Celera Annotation;CKLF-like MARVEL transmembrane domain containing 4	Cklfsf4;D19397;ENSMUSG00000051554;Gm9853	AF479815.1;BE952164.1;BC028852.1;CJ104384.1	74
Mm00801606_m1	Col4a5	collagen, type IV, alpha 5;mCG116862 Celera Annotation	-	AK080682.1;BC082788.1;AK133179.1;AB041350.1	77
Mm01290062_m1	Csf2	mCG13784 Celera Annotation;colony stimulating factor 2 (granulocyte-macrophage)	Csfgm;GMCSF;Gm-CSf;MGI-IGM	BC116880.1;X02333.1;X03019.1;X03221.1;M11848.1;BC116878.1;EU366957.1;AK053196.1	125
Mm00486849_m1	Ctla4	cytotoxic T-lymphocyte-associated protein 4;mCG113577 Celera Annotation	Cd152;Ctla-4;Ly-56	BC042741.1;AB097214.1;AK138891.1;BC052683.1;X05719.1	71
Mm02620111_s1	Cx3cr1	mCG14129 Celera Annotation;chemokine (C-X3-C motif) receptor 1	-	BC012653.1;AK137388.1;AK045634.1;AF102269.1;AK035902.1;AK140010.1;AK044557.1	107
Mm02620517_s1	Cxcr6	chemokine (C-X-C motif) receptor 6;mCG15845 Celera Annotation	BB217514;BONZO;STRL33	JC209414.1;BQ562712.1;AK052901.1;AK153914.1;BC096491.1;AK137703.1;AF305709.1	141
Mm00445235_m1	Cxcl10	chemokine (C-X-C motif) ligand 10;mCG12531 Celera Annotation	C7;CRG-2;INP10;IP-10;IP10;Ifi10;Scyb10;gIP-10;mob-1	M86829.1;AK151534.1;AK172540.1;BY165450.1;AK150380.1;AK151415.1;AK157139.1;AK157678.1;AK152814.1;AK157130.1;M33266.1;AK151210.1;AK150765.1;AK151248.1;AK157589.1;AK152568.1;AK144279.1;AF227743.1;BC030067.1;AK153181.1	59
Mm00444662_m1	Cxcl11	chemokine (C-X-C motif) ligand 11;mCG12536 Celera Annotation	Cxc11;H174;I-tac;Ip9;Itac;Scyb11;Scyb9b;b-R1;betaR1	AK040051.1;BC025903.1;AK020750.1;AF179872.1;AF178672.1;AF136449.1;AK050012.1	81
Mm00469712_m1	Cxcl16	mCG21161 Celera Annotation;chemokine (C-X-C motif) ligand 16	0910001K24Rik;AV290116;BB024863;CXCL16v1;CXCL16v2;SR-PSOX;Zmynd15;b2b498Clo	AK152547.1;AK153192.1;AK151330.1;AK028875.1;BC019961.1;AK030515.1;AK003085.1;AF301017.1;AK150224.1;AK152102.1;AF277001.1;AK009599.1	67
Mm00731329_s1	Cxcr1	mCG1035566 Celera Annotation;chemokine (C-X-C motif) receptor 1	Il8ra	AY749637.1;AY227797.1;AY390263.1	98
Mm99999054_s1	Cxcr3	chemokine (C-X-C motif) receptor 3;mCG2202 Celera Annotation	Cd183;Cmkar3	AB003174.1;AF045146.1;BC096626.1	57
Mm00434946_m1	Cxcl9	mCG12528 Celera Annotation;chemokine (C-X-C motif) ligand 9	BB139920;CMK;Mig;MuMIG;Scyb9;crg-10	AK157118.1;AK042480.1;M34815.1;AK156453.1;CT010194.1;AK156029.1;BC003343.1;AK157109.1;AK156012.1;AK042017.1	64

Mm00438656_m1	Edn1	endothelin 1;mCG19645 Celera Annotation	ET-1;preproET	D43775.1;BC029547.1;AK152647.1;U35233.1;AK153437.1;AB081657.1;AK040778.1	67
Mm01168928_g1	Elane	elastase, neutrophil expressed;mCG13398 Celera Annotation	Ela2;F430011M15Rik;NE	AK143710.1;BC145800.1	69
Mm00441238_m1	Ccl11	mCG8183 Celera Annotation;chemokine (C-C motif) ligand 11	Scya11;eotaxin	U26426.1;AW986913.1;AF128206.1;AF128205.1;AF128208.1;AF128207.1;AF128209.1;AK010146.1;BC027521.1;U40672.1;AK009307.1	78
Mm01204974_m1	Fas	mCG21463 Celera Annotation;Fas (TNF receptor superfamily member 6)	AI196731;APO1;APT1;CD95;TNFR6;Tnfrsf6;lpr	BC061160.1;M83649.1;DQ846748.1;AK002590.1	76
Mm00438864_m1	Fasl	Fas ligand (TNF superfamily, member 6);mCG22679 Celera Annotation	APT1LG1;CD178;CD95-L;CD95L;Fas-L;Faslg;Tnfsf6;gld	U10984.1;U06948.1;DQ846747.1;S76752.1;BC052866.1;AF288572.1;U58995.1;AK141268.1;AK040139.1	84
Mm01256744_m1	Fn1	fibronectin 1;mCG121782 Celera Annotation	E330027I09;Fn;Fn-1	AK133868.1;AK147683.1;BC004724.1;AK147249.1;AK147639.1;BC138421.1;BC145271.1;BC025521.1;AK147701.1;CN532664.1;AK159794.1;AK147663.1;AK090130.1;AK090135.1;S45680.1;AK170755.1;AK147315.1	58
Mm99999915_g1	Gapdh	mCG49966 Celera Annotation;mCG130520 Celera Annotation;mCG115100 Celera Annotation;mCG19641 Celera Annotation;mCG4178 Celera Annotation;mCG19420 Celera Annotation;mCG9609 Celera Annotation;mCG130428 Celera Annotation;mCG142399 Celera Annotation;mCG113945 Celera Annotation;mCG133580 Celera Annotation;mCG134295 Celera Annotation;mCG114112 Celera Annotation;mCG114986 Celera	Gapd	AK199549.1;BC085274.1;BC091768.1;AK191938.1;AK164415.1;AK140794.1;BC096590.1;BC085315.1;BC020407.1;BC095932.1;AK160399.1;BC145810.1;AK192040.1;BC023196.1;AK146435.1;BC092264.1;BC082592.1;AK147891.1;AK201736.1;BC093508.1;BC096440.1;BC092294.1;BU504528.1;AK189223.1;GU214026.1;M32599.1;BC083079.1;AK198590.1;BC085275.1;AK160753.1;BC083149.1;AK081405.1;BC083065.1;CJ155387.1;BC145812.1;AK144690.1;BC094037.1;AK147738.1;BC110311.1;BC083080.1;AK002273.1;BC092252.1	109
Mm01197698_m1	Gusb	mCG12423 Celera Annotation;glucuronidase, beta	AI747421;Gur;Gus;Gus-r;Gus-s;Gus-t;Gus-u;Gut;asd;g	AK151802.1;AK136519.1;AK159163.1;M28541.1;M63836.1;AK159804.1;AK041058.1;AK159564.1;AK152206.1;AK159526.1;BC071226.1;AK162436.1;M28540.1;AK150048.1;J03047.1;M19279.1	71
Mm00442834_m1	Gzmb	granzyme B;mCG130827 Celera Annotation	AI553453;CCP-1/C11;CCP1;Ctla-1;Ctla1;GZB	X04072.1;BC002085.1;M12302.1;CT010272.1;AK157856.1;AK167770.1	95
Mm00516005_m1	Hmox1	mCG14997 Celera Annotation;heme oxygenase 1	D8Wsu38e;HO-1;HO1;Hemox;Hmox;Hsp32	AK153566.1;AK154598.1;AK153516.1;AK159521.1;BY319630.1;AK150934.1;AK159959.1;AK151627.1;X13356.1;AK169227.1;M33203.1;BC010757.1;AK153420.1;AK151005.1;AK159870.1	69
Mm00497600_m1	Icos	mCG113578 Celera Annotation;inducible T cell co-stimulator	AILIM;CCLP;CRP-1;H4;Ly115	AB023132.1;BC034852.1;AJ250559.1;AK030827.1;AF257230.1	65
Mm01168134_m1	Ifng	interferon gamma;mCG1237 Celera Annotation	IFN-g;Ifg	FJ617516.1;BC119065.1;AK089574.1;BC119063.1;FJ617514.1;M28621.1;FJ617515.1;K00083.1	100
Mm00439620_m1	Il1a	mCG20997 Celera Annotation;interleukin 1 alpha	Il-1a	BC003727.1;AK156449.1;M14639.1;X01450.1	68

Mm00434228_m1	Il1b	mCG20999 Celera Annotation;interleukin 1 beta	IL-1beta;Il-1b	BC011437.1;M15131.1;AK168047.1;AK225002.1	90
Mm00446186_m1	Il1rn	interleukin 1 receptor antagonist;mCG4837 Celera Annotation	F630041P17Rik;IL-1ra	AK089222.1;AK155846.1;AK157157.1;M74294.1;AK076296.1;AK171385.1;AK155908.1;AK156034.1;BC042532.1;AK155263.1;M57525.1;AK154601.1;AF001795.1;AK155888.1;AK155756.1;M64404.1;S64082.1;AK155798.1;BY206809.1;AK170378.1;AK155641.1;AK171037.1	80
Mm00434256_m1	Il2	mCG12676 Celera Annotation;interleukin 2	Il-2	K02292.1;BC116845.1;U41504.1;X01772.1;U41506.1;U41505.1;AY147902.1;U41494.1;DQ836354.1;AF065915.1;AF065916.1;X73040.1;AF065914.1;BC116873.1	82
Mm01340213_m1	Il2ra	interleukin 2 receptor, alpha chain;mCG9569 Celera Annotation	CD25;Il2r;Ly-43	M54934.1;AK088594.1;M30856.1;K02891.1;AK037369.1;AK171665.1;BC114437.1	83
Mm00439631_m1	Il3	interleukin 3;mCG13767 Celera Annotation	BPA;Csfmu;HCGF;Il-3;MCGF;PSF	K01668.1;K01850.1;BC125552.1;A02046.1;BC125554.1;AK153634.1	85
Mm00445259_m1	Il4	interleukin 4;mCG13773 Celera Annotation	BSF-1;Il-4	AF352783.1;BC027514.1;X03532.1;M25892.1;AB174765.1;M13238.1	79
Mm00439646_m1	Il5	mCG13786 Celera Annotation;interleukin 5	Il-5	X04601.1;BC125366.1;BC132066.1;X06270.1	62
Mm00446190_m1	Il6	mCG11634 Celera Annotation;interleukin 6	Il-6	BC138766.1;BC145409.1;DQ788722.1;AK089780.1;AK150440.1;X06203.1;BC132458.1;X54542.1;J03783.1;AK152189.1	78
Mm01295803_m1	Il7	mCG8934 Celera Annotation;interleukin 7	A630026I06Rik;Il-7;hlb368	AK145184.1;BC110553.2;AK041307.1;AK052452.1;X07962.1;AK139858.1;AK040399.1	121
Mm01288386_m1	Il10	interleukin 10;mCG2645 Celera Annotation	CSIF;Il-10	M37897.1;BC120612.1;AK152344.1;BC137844.1	136
Mm01288989_m1	Il12b	mCG20097 Celera Annotation;interleukin 12b	Il-12b;Il-12p40;Il12p40;p40	AK162981.1;BC103614.1;AF128214.1;BC103609.1;AF128215.1;BC103608.1;BY154173.1;BC103610.1;M86671.1	63
Mm00434200_m1	Il12rb2	interleukin 12 receptor, beta 2;mCG130739 Celera Annotation	A930027I18Rik;IL-12RB2;Ifnm	AK040072.1;U64199.1;BC137745.1	74
Mm00434204_m1	Il13	interleukin 13;mCG13776 Celera Annotation	Il-13	M23504.1	56
Mm00434210_m1	Il15	interleukin 15;mCG119097 Celera Annotation	AI503618;IL-15	DQ083237.1;BC023698.1;DQ083236.1;AK085441.1;CJ137432.1;AK155616.1;U14332.1	73
Mm00439618_m1	Il17a	interleukin 17A;mCG7914 Celera Annotation	Ctla-8;Ctla8;IL-17;IL-17A;Il17	U43088.1;AK040420.1;BC119309.1;BC119303.1	80
Mm01274147_g1	Il18bp	mCG6766 Celera Annotation;interleukin 18 binding protein	IL-18BP;Igifbp;MC54L	AK003370.1;CT010334.1;CT010164.1;AK009877.1;AB019505.1;AK009721.1;AF122907.1;AF110802.1;AK008452.1;AF110803.1;BC018332.1	59
Mm00517640_m1	Il21	interleukin 21;mCG12674 Celera Annotation	IL-21	BC125416.1;AF254070.1;DQ645418.1;BC125414.1;AY428162.1	67
Mm00461162_m1	Il27	mCG145781 Celera Annotation;interleukin 27	IL-27;IL-27p28;Il30;p28	AY099297.1;AK152114.1;AK153466.1;BC119402.1	55
Mm01288580_m1	Irf1	interferon regulatory factor 1;mCG13768 Celera Annotation	AU020929;Irf-1	AK152104.1;CT010234.1;AY225160.1;BC003821.1;AK152005.1;AK155983.1;AK152193.1;M21065.1;AK153514.1;AK157347.1	66
Mm01222247_m1	Ikbkb	mCG6974 Celera Annotation;inhibitor of kappaB kinase beta	AI132552;IKK-2;IKK-beta;IKK2;IKK[b];IKKbeta	AK011359.1;AK151955.1;AK156294.1;AK139972.1;AK144603.1;AK153151.1;AK201390.1;AK155587.1;AK144048.1;AK151888.1;BC037723.1;AF088910.1;AK150884.1;AK086442.1;AF026524.1;AK182153.1;BC004772.1	63
Mm01324470_m1	Lcn2	mCG17450 Celera Annotation;lipocalin 2	24p3;AW212229;Sip24	BC132069.1;BC020275.1;BC132071.1;X14607.1;AK149774.1;S82469.1;AK002932.1	84
Mm00440228_gH	Lta	mCG15943 Celera Annotation;lymphotoxin A	LT;LT-[a];LT-alpha;LT[a];LTalpha;Ltx;TNF-beta;TNFSF1;Tnfb;Tnfsf1b;hlb382	M16819.1;BC099464.1;AK153694.1;AK156659.1;X14800.1;AK080308.1	59
Mm01298424_m1	Mpo	mCG119373 Celera Annotation;myeloperoxidase	mKIAA4033	AK220229.1;BC053912.1;X15313.1;AK160229.1;AY500847.1	63
Mm00440502_m1	Nos2	mCG1599 Celera Annotation;nitric oxide synthase 2, inducible	NOS-II;Nos-2;Nos2a;i-NOS;iNOS	AF065923.2;AF065921.2;AF065922.2;U43428.1;AY090567.1;M84373.1;M92649.1;M87039.1;AF065919.1;AF065920.1;BC062378.1	66
Mm00441242_m1	Ccl2	chemokine (C-C motif) ligand 2;mCG8184 Celera Annotation	AI323594;HC11;JE;MCAF;MCP-1;MCP1;SMC-CF;Scya2;Sigje	CT010187.1;BC145867.1;AF065931.1;AF065932.1;AK153520.1;AF065929.1;AF065930.1;AF065933.1;AK150937.1;BC055070.1;BC145869.1;AK132590.1;AK151789.1;AK153443.1;AK153468.1	74

Mm01617100_m1	Ccl12	mCG127264 Celera Annotation;chemokine (C-C motif) ligand 12;mCG123601 Celera Annotation	MCP-5;Scya12	AF065937.1;AF065938.1;U50712.1;U66670.1;AF065935.1;BC027520.1;AF065936.1;AK012356.1;AF065934.1	93
Mm00451734_m1	Pdcd1lg2	programmed cell death 1 ligand 2;mCG9110 Celera Annotation	B7-DC;Btdc;F730015O22Rik;PD-L2	AK089369.1;AF142780.2;AK141111.1;BC104139.1;BC104138.1;AK155002.1	95
Mm00451315_g1	Pf4	platelet factor 4;mCG1704 Celera Annotation	Cxcl4;Scyb4	AK160040.1;AK159278.1;AB017491.2;BC061111.1	66
Mm00812512_m1	Prf1	mCG15751 Celera Annotation;perforin 1 (pore forming protein)	Pfn;Pfp;Prf-1	M23182.1;X12760.1;X60165.1;BC137962.1;BC132300.1	95
Mm00478374_m1	Ptgs2	mCG5001 Celera Annotation;prostaglandin-endoperoxide synthase 2	COX2;Cox-2;PGHS-2;PHS-2;Pghs2;TIS10	M88242.1;BC052900.1;AK144726.1;AK144956.1;AK166221.1;M94967.1;AK172161.1;AK049923.1;M64291.1	80
Mm00442991_m1	Mmp9	matrix metallopeptidase 9;mCG17531 Celera Annotation	AW743869;B/MMP9;Clg4b;MMP-9;pro-MMP-9	Z27231.1;D12712.1;AK004651.1;AK159292.1;AK161176.1;X72795.1;BC046991.1;S67830.1;AK142787.1	76
Mm02342448_gH	Rps29	ribosomal protein S29;mCG2108 Celera Annotation;mCG7602 Celera Annotation	-	AK002939.1;AK012285.1;AK224925.1;BC024393.1;BC051203.1;L31609.1;AA217935.1	149
Mm00782550_s1	Socs1	suppressor of cytokine signaling 1;mCG126571 Celera Annotation	Cish1;Cish7;JAB;SOCS-1;SSI-1	BC132366.1;AK154706.1;AB000710.1;AF180302.1;AF120490.1;U88325.1;BC132368.1;AK028632.1;AB000677.1	88
Mm00850544_g1	Socs2	mCG4302 Celera Annotation;suppressor of cytokine signaling 2	8030460M17;AI527257;AW108012;CIS2;Cish2;D130043N08Rik;JAB;SOCS-2;SSI-2;hg	AK160537.1;AK137403.1;U88327.1;AK162591.1	137
Mm00450960_m1	Tbx21	mCG13239 Celera Annotation;T-box 21	TBT1;Tbet;Tblym	AF093099.1;BC137986.1;AF241242.1;AK156271.1;BC137988.1;AK054495.1	69
Mm00443258_m1	Tnf	mCG15911 Celera Annotation;tumor necrosis factor	DIF;TNF-a;TNF-alpha;TNFSF2;TNFalpha;Tnfa;Tnfsf1a	AK153319.1;AK153800.1;AK154223.1;AK155964.1;BC137720.1;M13049.1;M11731.1;AY423855.1;BY167004.1;BC117057.1;X02611.1	81
Mm00441236_m1	Ccl1	mCG8193 Celera Annotation;chemokine (C-C motif) ligand 1	BF534335;I-309;Scya1;Tca-3	BC120806.1;AF065924.1;M17957.1;M23501.1;AF065925.1;AF065926.1;AK019040.1	65
Mm00437136_m1	Tnfrsf18	tumor necrosis factor receptor superfamily, member 18;mCG23344 Celera Annotation	AITR;Gitr	AF229433.1;AF229434.1;U82534.1;AY157832.1;AY157833.1;AK081878.1;AK020762.1;AF229432.1	89
Mm00437214_m1	Tnfsf4	mCG9415 Celera Annotation;tumor necrosis factor (ligand) superfamily, member 4	Ath-1;Ath1;CD134L;OX-40L;Ox40l;TXGP1;Txgp1l;gp34	FJ176943.1;U12763.1	60
Mm00839222_m1	Tnfsf18	tumor necrosis factor (ligand) superfamily, member 18;mCG145597 Celera Annotation	Gitrl	BC137814.1;BC137815.1;AY320040.1;AY359852.1;AJ577579.1;AY234223.1;AJ577580.1	71
Mm00456355_m1	Tpo	mCG7809 Celera Annotation;thyroid peroxidase	-	X60703.1;AK047843.1	119
Mm01320970_m1	Vcam1	vascular cell adhesion molecule 1;mCG19764 Celera Annotation	CD106;Vcam-1	U12884.1;BC029823.1;AK016465.1;M84487.1;X67783.1;L08431.1;AK143401.1;AK030195.1;CT010373.1;BC011159.1;AK162954.1	71
Mm00437306_m1	Vegfa	mCG2676 Celera Annotation;vascular endothelial growth factor A	Vegf;Vpf	S37052.1;EF028705.1;AY750956.1;M95200.1;AY707864.1;S38100.1;S38083.1;AY750957.1;BQ832724.1;AY756068.1;BC061468.1;AY120866.1	61
Mm01295931_m1	Selp	mCG141166 Celera Annotation;selectin, platelet	CD62P;GMP-140;Grmp;LECAM3;PADGEM	BC109159.1;AK145335.1;M87861.1;M72332.1;BC109158.1;AK136243.1	67
Mm00445641_m1	Retn	mCG13014 Celera Annotation;resistin	ADSF;Fizz3;Rstn;Xcp4	BC051196.1;AF323080.1;AF290870.1;AK131613.1	80

Mm00441883_g1	Tnfrsf1a	mCG129428 Celera Annotation;tumor necrosis factor receptor superfamily, member 1a	CD120a;FPF;TNF-R;TNF-R-I;TNF-R1;TNF-R55;TNF-alphaR1;TNFAR;TNFR60;TNFRI;TNFRp55;TNFalpha-R1;Tnfr-2;Tnfr1;p55;p55-R	BC004599.1;AK154388.1;X57796.1;M59377.1;BC052675.1;AY541589.1;L26349.1;X59238.1;AK159989.1;M60468.1	82

Ninetytwo primers for genes of immunological interest plus three housekeeping genes and one control were purchased from Applied Biosystem. Assay ID, gene symbol(s), gene alias(es), gene bank mRNA, and amplicon length information were provided by the company; specific primers and probes sequences are proprietary information and were not provided by the company.

Supplementary Table 3: Primers for custom mouse PCR array #2

Assay ID	Gene Symbol(s)	Gene Name(s)	Gene Alias(es)	GenBank mRNA(s)	Amplicon Length
Mm00523597_m1	5430435G22Rik	RIKEN cDNA 5430435G22 gene	Rab7b	CJ137398.1;AK079999.1;AK030688.1;AK036056.1	105
Mm00502940_m1	Acer3	alkaline ceramidase 3	1110057L18Rik;5430429L08Rik;AV015045;Phca	BC023924.1;AK162403.1;AK080977.1;AK011668.1;AK004287.1;AK150629.1	105
Mm00479862_g1	Aif1	allograft inflammatory factor 1	AI607846;AIF-1;D17H6S50E;G1;Iba1	EF101555.1;AK006562.1;CT010265.1;D86382.1;BC021539.1;AF074959.1;AK006184.1;AB013745.1;CT010385.1	82
Mm00444767_m1	Aldh18a1	aldehyde dehydrogenase 18 family, member A1	2810433K04Rik;AI429789;Pycs	BC033427.1;AK166494.1;AK196084.1;AK153946.1;AK134148.1;AK159610.1;AF056573.1;AK166459.1;AF056574.1;AK168905.1;AK147711.1;AK151072.1;AK190906.1;BC037699.1	65
Mm00506949_m1	Arhgap4	Rho GTPase activating protein 4	A130009C12Rik;A530015A20Rik;Rgc1;c1;mKIAA0131	AK157294.1;AY183127.1;AY183124.1;AY183125.1;AY183126.1;BC109001.1;AY183128.1;AY183129.1;AK129060.1;BC109002.1;AF443826.1;AY183133.1	67
Mm01323072_m1	Arl11	ADP-ribosylation factor-like 11	ARLTS1;C730007L20Rik	AK008804.3;AK050053.1;BC064093.1	109
Mm00813516_m1	Arhgef18	rho/rac guanine nucleotide exchange factor (GEF) 18	AI467246;D030053O22Rik	BC060639.1;AK147923.1;AK041423.1	75
Mm00475988_m1	Arg1	arginase, liver	AI;AI256583;Arg-1;PGIF	U51805.1;CB947842.1;BC013341.1;BC050005.1;CT010173.1;AK149471.1	65
Mm00443898_m1	Atp2a3	ATPase, Ca++ transporting, ubiquitous	SERCA3b;Serca3	U49393.1;U49394.1;BC026147.1;BC017639.1;AK155904.1;AK089487.1	59
Mm01248177_m1	Aurka	aurora kinase A	AIRK1;ARK-1;AU019385;AW539821;Ark1;Aurora-A;Ayk1;IAK;IAK1;Stk6	AK169363.1;AY336976.1;BC005425.1;U69106.1;BC014711.1;AK145968.1;U80932.1;AK085861.1	69
Mm00492647_m1	Impact	impact, RWD domain protein	E430016J11Rik	AK138136.1;BC020524.1;D87973.1	99
Mm01218551_m1	Alox5ap	arachidonate 5-lipoxygenase activating protein	Flap	AK075967.1;BC026209.1;M96554.1;AK004002.1	60
Mm00479358_m1	Bcl11a	B cell CLL/lymphoma 11A (zinc finger protein)	2810047E18Rik;BCL-11A;Ctip1;D930021L15Rik;Evi9;Evi9a;Evi9b;Evi9c;mKIAA1809	AF186018.1;AK004395.1;AF169037.1;AF051525.1;AK043677.1;AK140949.1;AK045556.1;BC051418.1	79
Mm00805575_m1	Igf2bp2	insulin-like growth factor 2 mRNA binding protein 2	C330012H03Rik;IMP-2;Imp2;Neilsen	BC023758.1;AK049196.1;BU708344.1;AK077404.1;AK044984.1;AY531659.1;AK159654.1;BC054552.1	74
Mm00438023_m1	Casp1	caspase 1	ICE;Il1bc	AK132826.1;L03799.1;BC008152.1;L28095.1;AK163069.1	99
Mm00432307_m1	Casp4	caspase 4, apoptosis-related cysteine peptidase	CASP-11;CASP-4;Casp11;Caspl;ich-3	Y13089.1;U59463.1;BF018212.1;AK171877.1;AB480706.1;AK151547.1;BC061255.1;AK160328.1;AK156667.1	83
Mm00486784_m1	Cbfa2t3	core-binding factor, runt domain, alpha subunit 2, translocated to, 3 (human)	A630044F12Rik;AI465270;AW229127;Cbfa2t3h;ETO-2;Eto2;MTGR2	BC055951.1;AK041887.1;BC151156.1;AK033680.1;BC141396.1;AF038029.1	59
Mm00441242_m1	Ccl2	chemokine (C-C motif) ligand 2	AI323594;HC11;JE;MCAF;MCP-1;MCP1;SMC-CF;Scya2;Sigje	CT010187.1;BC145867.1;AF065931.1;AF065932.1;AK153520.1;AF065929.1;AF065930.1;AF065933.1;AK150937.1;BC145869.1;AK132590.1;BC055070.1;AK151789.1;AK153443.1;AK153468.1	74
Mm00443113_m1	Ccl7	chemokine (C-C motif) ligand 7	MCP-3;Scya7;fic;marc;mcp3	AF128194.1;AF128193.1;S50588.1;BC061126.1;S71251.1;AK078827.1;Z12297.1;L04694.1;BY163435.1	122

Mm00441260_m1	Ccl9	chemokine (C-C motif) ligand 9	CCF18;MRP-2;Scya10;Scya9	AF128196.1;CB950873.1;AK154511.1;AF128195.1;AF128198.1;AF128197.1;AF128200.1;AF128199.1;AF128202.1;AF128201.1;AF128204.1;BC139318.1;AF128203.1;AK155036.1;AK151649.1;U15209.1;AK151131.1;U19482.1;BC145962.1;AK154657.1;AK155032.1;U49513.1	93
Mm00438084_m1	Ccng1	cyclin G1	AI314029	AK143114.1;BC005534.1;AK153392.1;AK159429.1;AK128957.1;AK170397.1;AK167486.1;BC006609.1;AK167052.1;AK147026.1;Z37110.1;AK159831.1;AK160411.1;L49507.1	89
Mm01963251_s1	Ccr5	chemokine (C-C motif) receptor 5	AM4-7;CD195;Cmkbr5	AK155632.1;BG091923.1;AK154595.1;AK171391.1;D83648.1;AK155628.1	153
Mm99999114_s1	Ccr6	chemokine (C-C motif) receptor 6	CC-CKR-6;CCR-6;Cmkbr6;KY411	BC105669.1;BC100756.1;BC100757.1;BC100758.1;AK089712.1;AB016031.1;AB009369.1	151
Mm00839636_g1	Cd68	CD68 antigen	Lamp4;Scard1;gp110	X68273.1;AK002264.1;AW123877.1;AK170443.1;BC021637.1	86
Mm00444461_m1	Cd160	CD160 antigen	AU045688;By55	AK042093.1;DV660330.1;AK139789.1;AK145332.1;AF060982.1;BC021596.1	61
Mm00515420_m1	Cd19	CD19 antigen	AW495831	AK163608.1;AK089835.1;AK172008.1	69
Mm00515432_m1	Cd22	CD22 antigen	A530093D23;Lyb-8;Lyb8	AJ250680.1;BC150807.1;AJ250678.1;AJ250676.1;AK171589.1;L16928.1;AJ250683.1;AK041230.1;AJ250681.1;AF102134.1;AJ250679.1;AJ250677.1;AK172301.1;AF115400.1;AF115401.1;BC046957.1;AK143835.1;AK171990.1;AJ250684.1;AK156735.1;L02844.1	75
Mm00452054_m1	Cd274	CD274 antigen	A530045L16Rik;B7h1;Pdcd1l1;Pdcd1lg1;Pdl1	AK154403.1;BC066841.1;AF317088.1;AF233517.1;GQ904196.1	77
Mm00432423_m1	Cd79a	CD79A antigen (immunoglobulin-associated alpha)	Ig-alpha;Iga;Igalpha;Ly-54;Ly54;mb-1	X13450.1;AK143605.1	89
Mm00434143_m1	Cd79b	CD79B antigen	B29;Ig-beta;Igb;Igbeta	J03857.1;CT010358.1;AK143573.1;BC012226.1	74
Mm00650983_g1	Cdc20	cell division cycle 20	2310042N09Rik;C87100;p55CDC	AK160204.1;AK075998.1;AK168650.1;AK083459.1;AF312208.1;AK011723.1;AB045313.1;AK076030.1;BC003215.1;AK029424.1	156
Mm00496601_m1	Cdca3	cell division cycle associated 3	2410005A12Rik;C8;Grcc8;Tome-1	AK008606.1;AK011313.1;AK169792.1;BC027172.1;AK211930.1;AK010409.1;AK028159.1;BC002006.1;AK145537.1	87
Mm00788027_s1	Cdca7	cell division cycle associated 7	2310021G01Rik;AU044109;JPO1	AK075882.1;AK167734.1;AK011289.1;BC066169.1;AK028671.1	78
Mm01230623_g1	Cish	cytokine inducible SH2-containing protein	AI385595;CIS1;Cis;F17;F23	BC022178.1;AK170554.1;AK170877.1;AK171431.1;AK077338.1;BC003783.1;D31943.1	65
Mm00474134_m1	Clec2d	C-type lectin domain family 2, member d	Clr-b;Clrb;Ocil	HQ875062.1;AF321553.1;BC106776.1;HQ713439.1;AY320031.1;AF350409.1	92
Mm00501738_m1	Clec4d	C-type lectin domain family 4, member d	Clecsf8;Mpcl;mcl	AK155919.1;AK089500.1;BC099471.1;AF061272.1;AK150926.1;AK171409.1;AK170692.1	112
Mm00490934_m1	Clec4n	C-type lectin domain family 4, member n	Clec6a;Clecsf10;Nkcl	AF240358.1;AK131697.1;AK154673.1;AF240357.1;BC023008.1;AK171046.1	87
Mm00469582_m1	Cmpk2	cytidine monophosphate (UMP-CMP) kinase 2, mitochondrial	1200004E04Rik;TDKI;Tyki	AK150512.1;AK150885.1;BC048910.1;BC027329.1;BC057565.1;AK150725.1;AK004595.1;AK153469.1;AK150543.1;AK153429.1	77
Mm01306641_m1	Cnp	2',3'-cyclic nucleotide 3' phosphodiesterase	CNPase;Cnp-1;Cnp1	AK041969.1;AK158520.1;BC021904.1;AF332055.1;M31810.1;AK154654.1;AK158318.1;AK140280.1;BC005544.1;M58045.1;AF332056.1;AK172514.1;AK050628.1;AK172318.1	100
Mm00456011_m1	Ctsg	cathepsin G	-	BC125511.1;BC125513.1;X78544.1	70
Mm00484039_m1	Ctsk	cathepsin K	AI323530;MMS10-Q;Ms10q;catK	X94444.1;BC046320.1;AK132648.1;AK003425.1	73
Mm01255859_m1	Ctss	cathepsin S	-	Y18466.1;AK150274.1;AK153540.1;AK150758.1;AK028366.1;AJ002386.1;AK152618.1;BC002125.1;AF038546.1;AK150842.1;BC011104.1	75
Mm00469712_m1	Cxcl16	chemokine (C-X-C motif) ligand 16	0910001K24Rik;AV290116;BB024863;CXCL16v1;CXCL16v2;SR-PSOX;Zmynd15;b2b498Clo	AK152547.1;AK153192.1;AK151330.1;AK028875.1;BC019961.1;AK030515.1;AK003085.1;AF301017.1;AK150224.1;AK152102.1;AF277001.1;AK009599.1	67

Mm00434946_m1	Cxcl9	chemokine (C-X-C motif) ligand 9	BB139920;CMK;Mig;MuMIG;Scyb9;crg-10	AK157118.1;AK042480.1;M34815.1;AK156453.1;CT010194.1;AK156029.1;BC003343.1;AK157109.1;AK156012.1;AK042017.1	64
Mm00499348_m1	Cyp4f18	cytochrome P450, family 4, subfamily f, polypeptide 18	1810054N16Rik;Cyp4f3;Cypf18	AF233647.1;AK007863.1;BC013494.1	139
Mm00503627_m1	Dram1	DNA-damage regulated autophagy modulator 1	1200002N14Rik;Dram	AK151184.1;AK004552.1	141
Mm00624964_m1	E2f2	E2F transcription factor 2	E2F-2	AK087452.1;AK157676.1;BY231313.1;BC062101.1;AK157322.1	65
Mm00514634_m1	Ecm1	extracellular matrix protein 1	AI663821;p85	AK153471.1;L33416.1;BC145381.1;AK159334.1;BY224903.1;BC138693.1;AK169939.1;AK153009.1	66
Mm00472200_m1	Egln3	egl-9 family hypoxia-inducible factor 3	2610021G09Rik;AI505553;AI648162;Hif-p4h-3;Phd3;SM-20	BC044926.1;AK165972.1;AJ310548.1;AK170732.1;BC069893.1;AF421882.1;BC058278.1;AK044787.1	70
Mm01168928_g1	Elane	elastase, neutrophil expressed	Ela2;F430011M15Rik;NE	AK143710.1;BC145800.1	69
Mm00726459_s1	Gng2	guanine nucleotide binding protein (G protein), gamma 2	82	AK075698.1;AK158697.1;AK160396.1;AK003588.1;BC021599.1;AK036138.1;AK012405.1	92
Mm01310717_m1	Esd	esterase D/formylglutathione hydrolase	Es-10;Es10;FGH;sid478	AK088115.1;AK163074.1;AB025408.1;BC096644.1;AK010683.1;BC046766.1;AK021209.1;AK007527.1;AK079131.1	109
Mm00450997_m1	Gde1	glycerophosphodiester phosphodiesterase 1	1200003M13Rik;Mir16;RGS16	BC003902.1;AK004583.1;AK005361.1;AK150807.1;AF212860.1	75
Mm00512158_m1	Exoc3l4	exocyst complex component 3-like 4	1200009I06Rik;1600013K19Rik	AK005443.1;BC075664.1;BM247872.2;AK004674.1;BC042799.1	81
Mm00463693_m1	Fam102a	family with sequence similarity 102, member A	AI426465;C230093N12Rik;Eeig1	AK131145.1;AK157331.1;BC023752.1;AK170847.1;AK170951.1;AK170536.1;BC031157.1;AK157490.1;AK172536.1;AK049032.1	89
Mm00521600_m1	Fam129b	family with sequence similarity 129, member B	9130404D14Rik	AK153489.1;BY341437.1;AK046583.1;AK149818.1;AK170512.1;BC027843.1;AK051396.1;AK149888.1;AK150642.1;AK033735.1;AK152207.1;AK146953.1	87
Mm02601599_gH	Fbl	fibrillarin	AL022665;FIB;FLRN;RNU3IP1	Z22593.1;AK012620.1;BC092274.1;BC003813.1;BU511282.1;AK146328.1	63
Mm00505298_m1	Fblim1	filamin binding LIM protein 1	2410043F08Rik;Cal;Fblp1;Gt10;migfilin;migfilin(s)	AK170151.1;AK163587.1;BC004777.1;AK154680.1	85
Mm00442792_m1	Fcer2a	Fc receptor, IgE, low affinity II, alpha polypeptide	CD23;Fce2;Fcer2;Ly-42	X64224.1;X64223.1;BC119190.1;BC119164.1;S67074.1;M99371.1;AK172591.1;M34163.1;AY069980.1;AK156654.1;AY069981.1;S67073.1;S67072.1	68
Mm00519988_m1	Fcgr4	Fc receptor, IgG, low affinity IV	4833442P21Rik;CD16-2;FcgRIV;FcgammaRIV;Fcgr3a;Fcrl3	AK076532.1;AK170920.1;AF499613.1;BC027310.1;EU050648.1	69
Mm00520163_m1	Fcrla	Fc receptor-like A	BB219290;FCRL;FCRL1;FREB;Fcrlm1;Fcrx;Freb1;mFREB;mFcrX	AF426462.1;BC064708.1;AF531424.1;AF329487.1;AK041572.1	56
Mm00836315_g1	Fdps	farnesyl diphosphate synthetase	6030492I17Rik;AI256750;Fdpsl1;mKIAA1293	AK166026.1;AK151742.1;AK140881.1;BC087886.1;AK088601.1;CT010365.1;AK168008.1;BC048497.1;AF309508.1	175
Mm00516442_m1	Fgd2	FYVE, RhoGEF and PH domain containing 2	Tcd-2;Tcd2;ZFYVE4;tcs-2;tcs2	BC021845.1;AF017368.1;AK042260.1;AK156151.1;DQ344523.1	82
Mm00484464_s1	Fpr2	formyl peptide receptor 2	E330010I07Rik;Fpr-rs2	BC117066.1;AK054286.1	63
Mm01352550_g1	Gadd45g	growth arrest and DNA-damage-inducible 45 gamma	AI327420;C86281;CR6;DDIT2;OIG37	AK007410.1;AB021884.1;AF055638.1;BC001989.1;AK002237.1	58
Mm00444182_m1	Galk1	galactokinase 1	AA409894;GALK;Gk;Glk;Glk1	BC016602.1;AB027012.1;AK045694.1;AK011103.1;AK010340.1;BC050151.1;AK168716.1;AK088151.1	58

Mm00494576_g1	Gbp2	guanylate binding protein 2	-	AK150146.1;BC049141.1;CT010249.1;AF109168.1;BC011336.1;BC032882.1;AJ007970.1;AF077007.1	77
Mm00657086_m1	Gbp2b	guanylate binding protein 2b	Gbp-1;Gbp1;Mag-1;Mpa-1;Mpa1	M55544.1;BC108990.2;M63961.1;AK077796.1;EF494422.1	110
Mm00514996_m1	Gclm	glutamate-cysteine ligase, modifier subunit	AI649393;Gcmc;Glclr	AK176933.1;AK172387.1;U95053.1;AK028236.1;BC138783.1;BC132404.1;CT010256.1	76
Mm00457635_m1	Dnmt3l	DNA (cytosine-5-)-methyltransferase 3-like	D6Ertd14e;ecat7	AJ404467.1;EF051621.1;AF220524.1;EF051624.1;EF051623.1;BC083147.1;AK010434.1;EF051622.1	77
Mm00486029_m1	Glg1	golgi apparatus protein 1	AI593353;AW537898;CFR;CFR-1;ESL-1;MG-160;MG160;Selel	BC021306.1;X84037.1;AK154581.1	106
Mm00449274_m1	Mgll	monoglyceride lipase	AA589436;Magl;Mgl	BY706980.1;AJ316580.1;AK148242.1;AK185666.1;AJ001118.2;AK198283.1;AK131645.1;BY273849.1;AK208240.1;BC057965.1;AK182184.1;AK190239.1;AK198733.1;AK006949.1;AK197275.1;AK215869.1;AK148395.1;AK196912.1;AK206822.1;AK211363.1;AK188276.1;AK028008.1	78
Mm00497305_m1	Gpc1	glypican 1	AI462976	AF185613.1;AK154899.1;BC062902.1	96
Mm01328587_m1	Gpnmb	glycoprotein (transmembrane) nmb	DC-HIL;Dchil;ipd	AK171517.1;AK171465.1;AK159747.1;BC117855.1;AK159270.1;AK171347.1;AK171474.1;AF322054.1;AK171441.1;BC026375.1;AK154617.1;AK159908.1;AJ251685.1;AK159165.1;AK150739.1;AK170506.1;AK079220.1;AK149740.1;AK171618.1;AK171608.1;AK076347.1	66
Mm02620530_s1	Gpr84	G protein-coupled receptor 84	EX33	AF272948.1	139
Mm00442425_m1	Grk6	G protein-coupled receptor kinase 6	Gprk6	AK180303.1;AK161142.1;AF040747.1;AF040748.1;Y15799.1;Y15798.1;AF040749.1;Y15797.1;CA528447.1;AK146119.1;BC075719.1;Y15800.1;AK051405.1;BC057206.1	100
Mm01722569_g1	Gzmd	granzyme D	CCP5;Ctla-5;Ctla5	BC145434.1;BC141122.1;BC145433.1;AK131671.1;J03255.1	86
Mm00517945_m1	Haao	3-hydroxyanthranilate 3,4-dioxygenase	0610007K21Rik;0610012J07Rik;3-HAO;3-HAOxase;3HAO	BC094021.1;AK002295.1;AK171228.1;BC012872.1;AK002608.1	65
Mm01199527_s1	Hcar2	hydroxycarboxylic acid receptor 2	Gpr109a;Gpr109b;HM74;Niacr1;PUMA-G;Pumag;mHM74b	AK150795.1;BY516126.1;AK169956.1;AJ300198.1	66
Mm00727638_s1	Hilpda	hypoxia inducible lipid droplet associated	2310016C08Rik;AW049004;Hig2	AK009377.1;AW538932.1;AK144676.1;AF141311.1;BC083056.1	73
Mm00523556_s1	Hist1h3g	histone cluster 1, H3g	H3.1-221;M32460	BC125355.1;BC125357.1	93
Mm01341942_m1	Hk3	hexokinase 3	HK III;HK-III	AK159857.1;AK149651.1;BC028480.1;BC117861.1;AK171580.1;AK160063.1;AK160018.1;AK171248.1;AK140444.1;AK170813.1;BC117860.1;AK149992.1;AK159906.1;AK163658.1;AK154603.1;AK155701.1;AK171214.1	67
Mm00518151_m1	Hmgn3	high mobility group nucleosomal binding domain 3	1110002A15Rik;6330514M13Rik;BB071015;TRIP7	AK002970.1;AK003272.1;BC005693.1;AK153223.1	73
Mm00516005_m1	Hmox1	heme oxygenase 1	D8Wsu38e;HO-1;HO1;Hemox;Hmox;Hsp32	AK153566.1;AK154598.1;AK153516.1;AK159521.1;BY319630.1;AK150934.1;AK159959.1;AK151627.1;X13356.1;AK169227.1;M33203.1;BC010757.1;AK153420.1;AK151005.1;AK159870.1	69
Mm01168134_m1	Ifng	interferon gamma	IFN-g;Ifg	FJ617516.1;BC119065.1;AK089574.1;BC119063.1;FJ617514.1;FJ617515.1;M28621.1;K00083.1	100
Mm00492606_m1	Ifit2	interferon-induced protein with tetratricopeptide repeats 2	AV302338;Ifi54;P54	CJ044173.1;U43085.1;AK150253.1;BC050835.1;AK042366.1;AK038027.1;AK150206.1	66
Mm01704846_s1	Ifit3	interferon-induced protein with tetratricopeptide repeats 3	Ifi49;P49	BC003804.1;BC089563.1;AK151032.1;AK153226.1;L32974.1;AK077459.1	115
Mm01288386_m1	Il10	interleukin 10	CSIF;Il-10	M37897.1;BC120612.1;AK152344.1;BC137844.1	136

Mm00494938_m1	Il11ra1	predicted gene 2002;predicted gene 13305;interleukin 11 receptor, alpha chain 2;interleukin 11 receptor, alpha chain 1	OTTMUSG00000011351;ENSMUSG00000066201;Etl-2;Etl2;Il11ra-ps1;AI314697;GP130;Il-11ra;Il11ra;Il11ra2;NR1	AK159559.1;AK155407.1;U14412.1;BC004619.1;AK146742.1;AK143811.1;BC145341.1;X98519.1;BC138592.1;U69491.1;X74953.1;BC057664.1;BC069984.1;BC132549.1	64
Mm00434228_m1	Il1b	interleukin 1 beta	IL-1beta;Il-1b	BC011437.1;M15131.1;AK168047.1;AK225002.1	90
Mm00434295_m1	Il7r	interleukin 7 receptor	CD127;IL-7Ralpha	M29697.1;AK042264.1;AK041838.1;BC089571.1;AF078906.1;AK040740.1;BY776757.1;AK154516.1;AK040364.1	100
Mm01705338_s1	Isg15	ISG15 ubiquitin-like modifier	G1p2;IGI15;IP17;Irfp;UCRP	BC109346.2;CT010333.1;BC083156.1;X56602.1;BC031424.1	107
Mm00515213_m1	Itm2b	integral membrane protein 2B	AI256040;Bri;Bri2;Bricd2b;D14Sel6;E25BMM;imBRI2	AK160101.1;AK150396.1;BC004731.1;AK003928.1;AK159619.1;AK151889.1;AK159378.1;AB030203.1;AK002599.1;AK003224.1;AK153103.1;AK151714.1;AK005125.1;AK152516.1;AK077482.1;AK152983.1;AK152650.1;AK160725.1;AK159307.1;AK159962.1;AK152068.1;AK159792.1;AK003587.1;AK150378.1;U76253.1;AK159426.1;BC021786.1;AK159628.1;AK152731.1;AK159340.1;BC010320.1;AK076139.1;AK088270.1	80
Mm00439973_m1	Jak3	Janus kinase 3	fae;wil	L40172.1;AK164520.1;L32955.1;BC131647.1;AK142178.1;L33768.1;BC105577.1;AK156646.1;AK038268.1;AK088365.1;BC131646.1	66
Mm00521174_g1	Kif22	kinesin family member 22	AU021460;C81217;Kid;Kif22a	BC003427.1;AK084310.1;AK088033.1	118
Mm00441530_g1	Slpi	secretory leukocyte peptidase inhibitor	-	BC028509.1;U73004.1;U88093.1;AK010170.1;U94341.1	68
Mm00486279_m1	Soat1	sterol O-acyltransferase 1	8430426K15Rik;ACAT-1;AW550831;Acact;ald;hid	DQ903181.1;AK052299.1;S81092.1;BC083092.1;AK054149.1;AK142486.1;AK160017.1;AK159529.1;AK159393.1	78
Mm00495182_m1	Klrd1	killer cell lectin-like receptor, subfamily D, member 1	CD94	AF057714.1;AK136548.1;BC117112.1;AF039025.1;AF030312.1;BC120854.1;AF030311.1;AK162976.1	62
Mm00495267_m1	Lamp2	lysosomal-associated membrane protein 2	CD107b;LGP-B;Lamp II;Lamp-2;Lamp-2a;Lamp-2b;Lamp-2c;Mac3	BC138718.1;BY283193.1;AK088804.1;AK159272.1;BC138723.1;AK136550.1;J05287.1;AK146731.1;AK150766.1;AK163933.1;AK012149.1;AK159731.1;AK078193.1;AK048174.1;AK032974.1	90
Mm01324470_m1	Lcn2	lipocalin 2	24p3;AW212229;Sip24	BC132069.1;BC020275.1;BC132071.1;X14607.1;AK149774.1;S82469.1;AK002932.1	84
Mm00553642_m1	Lhfpl2	lipoma HMGIC fusion partner-like 2	6030465B15;AI447312;AW050335;mKIAA0206;vgim	AK087506.1;AK052969.1;AK031635.1;AK030103.1;BC040389.1	55
Mm00498820_m1	Lipa	lysosomal acid lipase A	AA960673;Lal;Lip-1;Lip1	AK154804.1;AK083099.1;Z31689.1;AK164007.1;AK153624.1;BC058564.1;AK088659.1;AK149791.1	85
Mm00521949_m1	Lmnb1	lamin B1	-	BC052729.1;BC058392.1;M35153.1;AK089110.1;X16705.1;AK079511.1;AK132252.1	79
Mm00434764_m1	Lpl	lipoprotein lipase	-	AK152238.1;AK151630.1;AK152035.1;AK151828.1;AK151772.1;AK151727.1;AK150328.1;AK152079.1;BC158040.1;AK151006.1;AK159268.1;M65258.1;AK045064.1;AK152706.1;AK151774.1;AK151801.1;AK151540.1;AK152350.1;AK002645.1;J03302.1;AK150691.1;AK150505.1;AK151434.1;AK152869.1;AK153428.1;AK152657.1;AK153006.1;AK151038.1;AK151195.1;AK151369.1;AK152400.1;AK150990.1;AK152956.1;AK151112.1;AK153242.1;AK151049.1;AK153425.1;AK150646.1;AK150532.1;AK151698.1;AK151216.1;BC003305.1;AK151679.1;AK151872.1;AK151521.1;AK150593.1;AK151563.1;AK150355.1;AK150984.1;AK152053.1;AK150577.1;AK152049.1;AK152390.1;AK150457.1;AK151093.1;AK152775.1;AK150672.1;CT010344.1;AK151893.1;AK150488.1;AK086023.1;AK079446.1;AK152251.1;AK150585.1;AK151105.1;AK151870.1;AK153148.1;AK152014.1;AK151866.1;AK170486.1;AK152733.1;AK152481.1;AK150375.1	61

Mm02620937_s1	P2ry6	pyrimidinergic receptor P2Y, G-protein coupled, 6	2010204J23Rik;P2Y6	BC064095.1;AK142680.1;BC027331.1;AX775901.1	117
Mm01275475_m1	Smox	spermine oxidase	B130066H01Rik;PAO;PAOh1;SMO	AK166350.1;AK159372.1;AJ567474.1;AF495852.1;AF495853.1;AJ567476.1;AF495851.1;AJ567478.1;AJ567480.1;AJ567473.1;AJ567475.1;BC004831.1;AJ567477.1;AK159275.1;AJ567479.1;AK143129.1	106
Mm04208404_m1	Lst1	leukocyte specific transcript 1	B144	U72643.1;AI323110.1;M18187.1;BC086881.1;AI182980.1	111
Mm00434787_m1	Ltf	lactotransferrin	Csp82;Lf;MMS10R;Ms10r	AK144556.1;BC008530.1;CT010339.1;FJ538998.1;AK150799.1;D88510.1;BC009662.1;AK151822.1;J03298.1;BC006904.1;AK036491.1;AK164781.1	67
Mm00657323_m1	Lyz1	lysozyme 1	Lyz;Lzp-s	BC061129.1;X51547.1	129
Mm01612741_m1	Lyz2	lysozyme 2	AI326280;Lys;Lysm;Lyzs;Lzm;Lzm-s1;Lzp	BC054463.1;AK148516.1;AK153475.1;AK159276.1;AK159640.1;AK153244.1;BC002069.1;AK150998.1	134
Mm00712529_m1	Mcm10	minichromosome maintenance deficient 10 (S. cerevisiae)	2410041F14Rik;AU018508;C330019M07Rik;C79164	AK030780.1;BC023236.1;BC120689.1;BC064314.1;BC094576.1;BC120687.1	66
Mm00484848_m1	Mcm6	minichromosome maintenance deficient 6 (MIS5 homolog, S. pombe) (S. cerevisiae)	ASP-l1;D1Wsu22e;Mcmd6	AK180079.1;AK150726.1;D86726.1;AK145520.1;BC057584.1;AK146770.1;AK194535.1;AK146896.1;BC050886.1;AK088493.1;BC051658.1	89
Mm01233136_m1	Mdm2	transformed mouse 3T3 cell double minute 2	1700007J15Rik;AA415488;Mdm-2	BC050902.1;AK150530.1;X58876.1;CN703560.1;AK152685.1;AK160069.1;AK168690.1;AK004881.1;BC092270.1;AK004719.1;AK088638.1;U47934.1	113
Mm00484956_g1	Mef2b	myocyte enhancer factor 2B	AI451606	BC045147.1;D87836.1;D87835.1;D87834.1;D87833.1;CO811773.1	60
Mm00434920_m1	Mertk	c-mer proto-oncogene tyrosine kinase	Eyk;Mer;Nyk;nmf12	AK029009.1;AK030158.1;L11625.1;U21301.1	55
Mm00498294_m1	Mgst1	microsomal glutathione S-transferase 1	1500002K10Rik;Gst	AF159050.1;AK005122.1;BC009155.1;AK002800.1	74
Mm01298424_m1	Mpo	myeloperoxidase	mKIAA4033	AK220229.1;BC053912.1;X15313.1;AK160229.1;AY500847.1	63
Mm00491795_m1	Mrps22	mitochondrial ribosomal protein S22	3100002P07Rik;Rpms22	AK003836.1;BC051198.1;AK013925.1;AK154600.1	113
Mm00442778_m1	Ms4a2	membrane-spanning 4-domains, subfamily A, member 2	FcRB;Fce1b;Fcer1b;Fcrbeta;Ms4a1;fcERI	BC139323.1;AK161121.1;J05019.1;CN663232.1;BC139324.1;AK144021.1;BB847820.1	65
Mm00496660_g1	Mt1	metallothionein 1	MT-I;Mt-1	AK018727.1;S62785.1;AA050166.1;AK011040.1;AK007983.1;BC109369.1;BC036990.1	88
Mm01320970_m1	Vcam1	vascular cell adhesion molecule 1	CD106;Vcam-1	U12884.1;BC029823.1;AK016465.1;M84487.1;X67783.1;AK030195.1;CT010373.1;L08431.1;BC011159.1;AK162954.1;AK143401.1	71
Mm00450101_m1	Nap1l1	nucleosome assembly protein 1-like 1	AA407126;AI256722;D10Ertd68e;NAP-1	X61449.1;BC076591.1;AK169301.1;AK012250.1;AK145766.1;D12618.1;AK050375.1;AK136161.1;AK153531.1;AK004633.1;AK152011.1;AK007322.1;AK216281.1;AK032896.1	71
Mm00626772_m1	Nceh1	neutral cholesterol ester hydrolase 1	Aadacl1;B230106I24Rik;CPO-BP;Nceh	AK173158.1;AK135837.1;AK045363.1;BC082569.1;AK085767.1;AK128968.1;AK034339.1	67
Mm01250218_m1	Ngp	neutrophilic granule protein	bectenecin	AK009649.1;BC119403.1;U95002.1;AK165210.1;BC119405.1	66
Mm00479705_m1	Nop58	NOP58 ribonucleoprotein	MSSP;Nol5;SIK;nop5	AF053232.1;BC085135.1;BC076604.1;AK134286.1;AK133959.1;AK044216.1	74
Mm00506520_m1	Nrm	nurim (nuclear envelope membrane protein)	2610307M02Rik;AI429796	BC026576.1;BC021750.1	86
Mm00497713_m1	Nup210	nucleoporin 210	9830001L10;AI836801;Pom210;gp190;gp210	AF113751.1;AK173056.1;AK053417.1;AK157272.1;BC052468.1	138
Mm00505601_m1	Nusap1	nucleolar and spindle associated protein 1	2610201A12Rik;AI481307;ANKT;AW547774;BB165529;BM037;LNP;NuSAP;Q0310;SAPL	BC100392.1;AK044819.1;AK042697.1;AK076028.1;BC004787.1;AK088389.1;AF305710.1	58
Mm01952477_s1	P2ry14	purinergic receptor P2Y, G-protein coupled, 14	A330108O13Rik;Gpr105;P2Y14	AK140301.1;BC028995.1;AK039791.1;AK155035.1;AK164982.1;BC058558.1;AF177211.1	97

Mm00440502_m1	Nos2	nitric oxide synthase 2, inducible	NOS-II;Nos-2;Nos2a;i-NOS;iNOS	AF065923.2;AF065921.2;AF065922.2;M84373.1;U43428.1;M92649.1;M87039.1;AF065919.1;AY090567.1;AF065920.1;BC062378.1	66
Mm00470315_m1	Cmtm3	CKLF-like MARVEL transmembrane domain containing 3	9430096L06Rik;AI413895;BNAS2;Cklfsf3	AK003882.1;AK079165.1;AY241870.1;AK172091.1;BC145817.1;BC145819.1;BC003230.1	67
Mm00517793_m1	Pbk	PDZ binding kinase	2810434B10Rik;AW538537;D14Ertd732e;TOPK	AB041882.1;AK076121.1;BC020099.1;AK003838.1	103
Mm00834521_g1	Pdlim1	PDZ and LIM domain 1 (elfin)	CLP36;Clim1;mClim1	AK158992.1;AK088089.1;AF053367.2;AK158173.1;AK146399.1;AK076285.1;AK146225.1;BC004809.1	70
Mm01348912_g1	Pdpn	podoplanin	Gp38;OTS-8;RANDAM-2;T1-alpha;T1a;T1alpha	CX240531.1;AK158855.1;AJ250246.1;BC026551.1	120
Mm00451315_g1	Pf4	platelet factor 4	Cxcl4;Scyb4	AK160040.1;AK159278.1;AB017491.2;BC061111.1	66
Mm00449846_m1	Phlda3	pleckstrin homology-like domain, family A, member 3	Tih1	BC023376.1;AK210974.1;AK003767.1;BC023408.1;AK199905.1;AK214255.1;AK206046.1	128
Mm00659576_m1	Pik3cb	phosphatidylinositol 3-kinase, catalytic, beta polypeptide	1110001J02Rik;AI447572;p110beta	BC127073.1;AK154106.1;AK165230.1;AK090116.1;AK081350.1;AK003230.1	142
Mm00479105_m1	Pla2g7	phospholipase A2, group VII (platelet-activating factor acetylhydrolase, plasma)	R75400	AK155678.1;AK051454.1;AK005158.1;U34277.1;AK005210.1	111
Mm00475794_m1	Plin2	perilipin 2	AA407157;ADPH;Adfp;Adrp	AK167140.1;AK152896.1;AK152081.1;AK090277.1;AK150177.1;BC047124.1;AK153222.1;M93275.1;AK154582.1;AK152263.1;AK078967.1;AY035850.1;AY035851.1;AK011014.1;AK152877.1;AK002911.1;BC054766.1;AK152982.1	79
Mm00440924_g1	Plk1	polo-like kinase 1	Plk;STPK13	U01063.1;AK148050.1;BC006880.1;AK148076.1;L19558.1;AK134045.1;AK164027.1	92
Mm00448326_m1	Pou2af1	POU domain, class 2, associating factor 1	BOB.1;Bob-1;Bob1;OBF-1;OBF.1;OCA-B;OCAB	AK135080.1;U43788.1;BC058611.1;Z54283.1;AK088587.1	119
Mm00504516_m1	Ppap2b	phosphatidic acid phosphatase type 2B	1110003O22Rik;2610002D05Rik;AV025606;D4Bwg0538e;D4Bwg1535e;Lpp3;Ppab2b	AK076183.1;AK160056.1;AK159136.1;AK011276.1;BC005558.1	105
Mm00478323_m1	Prtn3	proteinase 3	PR3;mPR3	U43525.1;U97073.1	70
Mm00650862_m1	Ranbp1	RAN binding protein 1	Htf9a	BC061140.1;X56045.1;AK002989.1;AK088781.1;AK008276.1;L25255.1;AK151167.1;AK153074.1;AK166642.1;X56046.1;AK167053.1	145
Mm00450170_m1	Rgs1	regulator of G-protein signaling 1	BL34	AK170366.1;AK154394.1;AK171306.1;AK138688.1;AK089293.1;AK170957.1;BC028634.1;BC052684.1;AF215667.1	117
Mm01623058_s1	Rps24	ribosomal protein S24	-	AK010856.1;AK049387.1	115
Mm01180369_g1	Rps27a	ribosomal protein S27A	0610006J14Rik;Uba52;Ubb;Ubc	BC096392.1;BC081446.1;AK018706.1;BC002108.1	66

Mm00491265_m1	Rsad2	radical S-adenosyl methionine domain containing 2	2510004L01Rik;Vig1;cig5	AK153234.1;AK153003.1;AK153556.1;AK150731.1;AK151339.1;AK153306.1;AK153232.1;AK152601.1;AK036373.1;AK152235.1;AK152050.1;AK151412.1;AK151538.1;AK153078.1;AK152493.1;AK152664.1;AK152310.1;AK153024.1;AK152895.1;AK152600.1;AK151140.1;AF442152.1;AK150406.1;AK151476.1;AK152644.1;AK152796.1;AK152065.1;AK151846.1;AK152075.1;AK152962.1;AK151971.1;AK151191.1;AK151502.1;AK151909.1;AK150525.1;AK151767.1;AK151906.1;AK153077.1;AK150812.1;AK152861.1;AK153435.1;AK151833.1;AK152934.1;AK151954.1;AK151308.1;AK150767.1;AK151825.1;AK150783.1;AK151414.1;AK010906.1;AK149900.1;AK150425.1;AK151286.1;AK150788.1;AK150616.1;AK153350.1;AK151410.1;AK150069.1;BC057868.1;AK150684.1;AK151206.1;AK153062.1;AK151185.1;AK152631.1;CT010378.1;AK153318.1;AK151770.1;AK152354.1;AK152239.1;AF236064.2;AK153324.1;AK152880.1;AK150536.1;AK159147.1;AK152241.1;AK151940.1;AK150835.1;AK152701.1;AK151389.1	70
Mm00656925_m1	S100a9	S100 calcium binding protein A9 (calgranulin B)	60B8Ag;AW546964;BEE22;Cagb;GAGB;L1Ag;MRP14;p14	BC027635.1;M83219.1;AK143826.1;BY112406.1	162
Mm00441203_m1	Saa3	serum amyloid A 3	AV098916;Saa-3;l7R3	BC055885.1	63
Mm00612478_s1	Samd9l	sterile alpha motif domain containing 9-like	AA175286;ESTM25;mKIAA2005	AK032210.1;AK173329.1;AK035419.1;AK172649.1;BC031151.1;BC079894.1;AK052535.1	126
Mm00455133_m1	Sbk1	SH3-binding kinase 1	Sbk	BC024114.1;BC031759.1;BC025837.1	120
Mm00489742_m1	Sdcbp	syndecan binding protein	MDA-9;Sycl;syntenin-1	AK152017.1;AK150321.1;AK159677.1;AF003693.1;AK178827.1;AK150661.1;AK152080.1;AK153042.1;AK169219.1;AK150801.1;AK152552.1;AK168004.1;AK206044.1;AK159124.1;AK152577.1;AK152109.1;BC019400.1;AK150736.1;AK033412.1;AK150623.1;AK153112.1;AK152001.1;AK150721.1;AK168777.1;AK167513.1;AK169415.1;AF077527.1;AK028429.1;AK152434.1;AK150278.1;AK169687.1;AK159538.1;AK146085.1;AK165657.1;AK150707.1;AK150996.1;AK171162.1;AK152855.1;AK159323.1;AK212103.1	77
Mm00441361_m1	Sema7a	sema domain, immunoglobulin domain (Ig), and GPI membrane anchor, (semaphorin) 7A	2900057C09Rik;CDw108;H-Sema-L;M-Sema-L;Semal	AB017532.1;AF176670.1;AF030699.1;AK156123.1;BC057875.1	119
Mm01179680_m1	Serinc1	serine incorporator 1	1500011D18Rik;AI315070;AIGP-2;TMS-2;Tde1l;Tde2;Tms2;mKIAA1253	AK215179.1;AK078632.1;AK133668.1;AK167817.1;AK205768.1;AF181685.1;AK217736.1;AK133975.1;AK141574.1;AK159280.1;AK159436.1;AK088340.1;BC006940.1;AK208925.1;AK005203.1;AK148745.1;AK135359.1;AK209793.1;BC017148.1;AB029500.1;AK002847.1	70
Mm00505954_m1	Sf3b3	splicing factor 3b, subunit 3	1810061H24Rik;5730409A01Rik;AA409318;D8Ertd633e;RSE1;SAP130;mKIAA0017	AK088268.1;BC042580.1;AK196304.1;AK164199.1;AK193615.1;AK147914.1;AK046845.1;AK128988.1;AK196066.1;BC011412.1;BC139015.1;BC139016.1;AK040625.1;AK129035.1	64
Mm01293286_m1	Slamf8	SLAM family member 8	5830408F06Rik;Blame;SBBI42	AK017911.1;AK156077.1;AB196831.1;AK142633.1;AB196830.1;AB196829.1;BC024587.1;AB196828.1	99
Mm00443045_m1	Slc11a1	solute carrier family 11 (proton-coupled divalent metal ion transporters), member 1	Bcg;Ity;Lsh;Nramp;Nramp1;ity	AK171343.1;X75355.1;BC109138.1;AK171393.1;L13732.1;AK144237.1;BC109137.1;AK090303.1	60
Mm00554217_m1	Slc2a6	solute carrier family 2 (facilitated glucose transporter), member 6	A330096C23;F630103L12Rik;Glut6;Glut9	BC141168.1;AK150104.1;BC145480.1;BC058210.1;AK172411.1;AK163445.1;AK079650.1;AK167904.1;AK137441.1;AK089246.1;AK166312.1	64
Mm01254822_m1	Slc40a1	solute carrier family 40 (iron-regulated transporter), member 1	Dusg;Fpn1;IREG1;MTP;MTP1;Ol5;Pcm;Slc11a3;Slc39a1	AK008700.1;AF226613.1;BC003438.1;AF231120.1;AF215637.1;AK032732.1;AK167608.1;AK159294.1;AK159855.1;AK147137.1;AK144780.1	63

Mm00506023_m1	Slc6a8	solute carrier family 6 (neurotransmitter transporter, creatine), member 8	AA589632;CRT;CRTR;CT1;CTR5;Creat	AB077327.1;BC049801.1;AF459435.1;BC145319.1;BC141067.1	68
Mm00442530_m1	Slc7a11	solute carrier family 7 (cationic amino acid transporter, y+ system), member 11	9930009M05Rik;AI451155;sut;xCT	AK171192.1;AK089202.1;AK171252.1;BC141408.1;BC141402.1;AK162233.1;AY766237.1;AY766236.1;AB022345.1;AK165599.1;AK037490.1;AK037742.1;AK163168.1	66
Mm00441516_m1	Slc7a5	solute carrier family 7 (cationic amino acid transporter, y+ system), member 5	4F2LC;D0H16S474E;LAT1;TA1	AB017189.1;AK148134.1;AK204407.1;BC026131.1;AK190487.1;AK142288.1;AK207173.1;AB023409.1;AK178111.1	86
Mm01185477_m1	Smap2	small ArfGAP 2	1810031K02Rik;AA408614;Smap1l	BC031581.1;BC032217.1;BC052413.1	58
Mm00476227_m1	Anpep	alanyl (membrane) aminopeptidase	AP-M;AP-N;Apn;Cd13;P150	BC017011.1;AK171427.1;BC005431.1;BC040792.1;AK143746.1;U77083.1	63
Mm00511052_g1	Snx10	sorting nexin 10	2410004M09Rik	AK150999.1;CT010276.1;AK150704.1;AK166121.1;AK150878.1;AK151320.1;AK169110.1;AK152004.1;AK153356.1;AK151520.1;AK153127.1;AK152825.1;BC010334.1;AK028163.1;AK010399.1	90
Mm01152449_g1	Srsf1	serine/arginine-rich splicing factor 1	1110054N12Rik;5730507C05Rik;6330415C05Rik;AI482334;AW491331;Asf;Sf2;Sfrs1	BC046773.1;AK169623.1;BY743476.1;AK150535.1;BC058627.1;CF739274.1;AK169203.1;AK078715.1;AK018176.1;AK053674.1;X66091.1	59
Mm00769566_m1	Srxn1	sulfiredoxin 1 homolog (S. cerevisiae)	1700127B04Rik;AI854065;AW488194;Npn3;Srx;Srx1;TX01	AK007296.1;BC011325.1;AK161066.1;BC049957.1;AK159613.1;AK159226.1;AK089976.1	87
Mm00486119_m1	St6gal1	beta galactoside alpha 2,6 sialyltransferase 1	AW742324;Siat1;St6Gal-I;St6gal;St6galI	CF741963.1;BC027833.1;AK084124.1;D16106.1;BC092222.1;BC096026.1	95
Mm00600456_m1	Tipin	timeless interacting protein	1110005A05Rik;1110018P21Rik	AK003802.1;AK003451.1;AK011357.1;BC016211.1;AK038111.1	78
Mm01246403_g1	Tk1	thymidine kinase 1	D530002A18Rik;Tk-1;Tk1a;Tk1b	AK139244.1;AK085188.1;AK087988.1;CF732600.1;BC091758.1;BC012215.1;M11945.1;M19438.1;S57244.1	91
Mm01233819_m1	Tlr13	toll-like receptor 13	AI666735;Gm713	BC117913.2;AY510706.1;AK155696.1;AK170044.1;BC117914.2	66
Mm00442346_m1	Tlr2	toll-like receptor 2	Ly105	BY743020.1;AY179346.1;AF185284.1;AF216289.1;AK154504.1;AK005043.1;AF124741.1;AF165189.1;BC014693.1	69
Mm00447578_m1	Tnfaip2	tumor necrosis factor, alpha-induced protein 2	B94;Exoc3l3;M-sec;Tnfip2;tnfb94	BC138106.1;L24118.1;AK087680.1;BC138107.1;AK170719.1;AK154405.1;AK172653.1	82
Mm04209424_g1	Trem2	triggering receptor expressed on myeloid cells 2	TREM-2;Trem2a;Trem2b;Trem2c	BC033485.1;AY024348.1;AK155163.1;AK171358.1;BC052784.1;BC032959.1;AF213458.1;AK039477.1;AY187009.1	90
Mm00446715_m1	Trf	transferrin	AI266983;Cd176;HP;Tf;Tfn;hpx	AK142599.1;AK168368.1;BC020295.1;BC058216.1;BC018573.1;BC008559.1;AK149559.1;AK168938.1;BC012313.1;AK149595.1;AK150782.1;AK168405.1;AK085754.1;BC022986.1;AK146549.1;AF440692.1;BC092046.1;AK168419.1;BC058218.1;AK169187.1	62
Mm00847804_gH	Tubb4b	zinc finger protein 646;tubulin, beta 4B class IVB	6820429M01;4930542G03Rik;Tubb2c;Tubb2c1	AK194925.1;AK167022.1;BC098458.1;BC022919.1;BC083319.1;AK015901.1;AK076895.1;AK214079.1;BC005547.1;AK002568.1;CJ309661.1;AK146587.1	144
Mm00493988_m1	Uba1	ubiquitin-like modifier activating enzyme 1	A1S9;Sbx;Ube-1;Ube1x	AK088057.1;D10576.1;AK143416.1;AK081808.1;AK219392.1;BC138200.1;BC145984.1;BC058630.1;AK171667.1;X62580.1;AK088528.1	71
Mm01972246_s1	Ubd	ubiquitin D	FAT10	AK008552.1	170
Mm00436987_m1	Tgm2	transglutaminase 2, C polypeptide	G[a]h;TG2;TGase2;tTG;tTGas	AK143712.1;AK080224.1;M55154.1;AK089481.1;AK159255.1;AK080593.1;AK052912.1;AK168990.1;AK086001.1;AF076928.1;AK152152.1;AK152627.1;AK151776.1;AK169356.1;BC016492.1;AF114266.1	72

Mm01188805_m1	Usp18	ubiquitin specific peptidase 18	1110058H21Rik; AW047653;UBP43;Ubp15	AK149772.1;AF069502.1;BC138577.1;AK151011.1;AK004296.1;AK152574.1;AK151260.1;BC138562.1;CT010228.1;AK152034.1;AK151722.1;AK153312.1	65
Mm00841195_m1	Vars2	valyl-tRNA synthetase 2, mitochondrial (putative)	1190004I24Rik;Vars2l;mKIAA1885	AK038117.1;AK155386.1;AK076629.1;AK173288.1;AK159301.1; AK129008.1;BC057036.1	68
Mm01333430_m1	Vim	vimentin	-	BC089335.1;AK213831.1;AK203121.1;AK151196.1;AK205443.1;AK159539.1;X56397.1;AK203726.1;AK169181.1;AK076001.1;X51438.1;AK150270.1;AK133726.1;AK153019.1;M24849.1;M26251.1;AK012469.1;AK170429.1	62
Mm00494160_m1	Vpreb3	pre-B lymphocyte gene 3	8HS-20;AI528709;Vpreb-3	BC062250.1;AK008794.1	63
Mm00435379_m1	Nrp1	neuropilin 1	C530029I03;NP-1;NPN-1;Npn1;Nrp	AK149752.1;AK188315.1;AK141964.1;AK192303.1;AK148372.1; AK159219.1;D50086.1;BC060129.1	57
Mm99999915_g1	Gapdh	glyceraldehyde-3-phosphate dehydrogenase	Gapd	AK199549.1;BC092264.1;BC082592.1;AK147891.1;BC085274.1;BC091768.1;AK201736.1;AK191938.1;BC093508.1;BC096440.1;BC092294.1;BU504528.1;AK189223.1;AK164415.1;BC096590.1;GU214026.1;AK140794.1;BC085315.1;M32599.1;BC083079.1;AK198590.1;BC020407.1;BC085275.1;AK160753.1;BC083149.1;BC095932.1;AK081405.1;BC083065.1;AK160399.1;BC145812.1;AK144690.1;CJ155387.1;BC145810.1;BC094037.1;AK192040.1;AK147738.1;BC110311.1;AK002273.1;BC023196.1;BC083080.1;BC092252.1;AK146435.1	109
Mm01197698_m1	Gusb	glucuronidase, beta	AI747421;Gur;Gus;Gus-r;Gus-s;Gus-t;Gus-u;Gut;asd;g	AK151802.1;AK136519.1;AK159163.1;M28541.1;AK159804.1;M63836.1;AK041058.1;AK159564.1;AK152206.1;AK159526.1;BC071226.1;AK162436.1;M28540.1;AK150048.1;J03047.1;M19279.1	71
Mm02342448_gH	Rps29	ribosomal protein S29	-	AK002939.1;AK012285.1;AK224925.1;BC024393.1;BC051203.1;L31609.1;BC055750.1;AA217935.1	149

188 primers including three housekeeping genes were purchased from Applied Biosystem as described in Material and Methods. Assay ID, gene symbol(s), gene alias(es), gene bank mRNA, and amplicon length information were provided by the company; specific primers and probes sequences are proprietary information and were not provided by the company.

Supplementary Table 4. Primers for custom rat PCR array

Assay ID	Gene Symbol(s)	Gene Name(s)	Gene Alias(es)	GenBank mRNA(s)	Amplicon Length
Rn01775763_g1	Gapdh	glyceraldehyde-3-phosphate dehydrogenase	BARS-38;Gapd	AB017801.1;M17701.1;BC087743.1;M29341.1;X02231.1;M11561.1;BC059110.1	174
Rn00566655_m1	Gusb	glucuronidase, beta	Ac2-223;Gus-s	M13962.1;Y00717.1	63
Rn00820645_g1	Rps29	ribosomal protein S29	-	FQ223975.1;FQ221523.1;FQ216880.1;FQ228434.1;FQ224820.1;FQ209501.1;FQ221661.1;FQ217213.1;FQ223378.1;FQ222368.1;FQ217140.1;FQ222293.1;X59051.1;FQ222143.1;FQ223222.1;FQ221457.1;FQ224661.1;FQ224465.1;FQ221640.1;FQ221587.1;FQ223289.1;BC058150.1;FQ221360.1;FQ223885.1;FQ218769.1;FQ211740.1;FQ221707.1;FQ228730.1;FQ224325.1;FQ222711.1;FQ221030.1;FQ222566.1;FQ223071.1;FQ224400.1;FQ221875.1;FQ210507.1;FQ218037.1;FQ224476.1;FQ221307.1;FQ218006.1;FQ222937.1;FQ221298.1;FQ221942.1;FQ221251.1	73
Rn99999125_m1	Bcl2	B-cell CLL/lymphoma 2	Bcl-2	L14680.1;AF512835.1	104
Rn00566466_m1	C3	complement component 3	-	X52477.1;CO796620.1	72
Rn00671924_m1	Ccl4	chemokine (C-C motif) ligand 4	Mip1-b;Scya4	EF121997.1;U06434.1;EF121996.1	68
Rn00579590_m1	Ccl5	chemokine (C-C motif) ligand 5	Rantes;Scya5	U06436.1;EF122007.1;BC058138.1;EF121972.1	80
Rn01467286_m1	Ccl7	chemokine (C-C motif) ligand 7	-	-	77
Rn01439563_m1	Ccl19	chemokine (C-C motif) ligand 19	-	-	62
Rn00571950_s1	Ccr1	chemokine (C-C motif) receptor 1	-	BC079207.1	96
Hs99999901_s1	18s rRNA	-	-	-	0
Rn01637698_s1	Ccr2	chemokine (C-C motif) receptor 2	-	-	103
Rn02134292_s1	Ccr3	chemokine (C-C motif) receptor 3	Cmkbr3	Y13400.1	80
Rn02132969_s1	Ccr5	chemokine (C-C motif) receptor 5	Ckr5;Cmkbr5	BC078756.1	81
Rn01536609_m1	Cmtm3	CKLF-like MARVEL transmembrane domain containing 3	Cklfsf3	BC169107.1	72
Rn01456850_m1	Csf2	colony stimulating factor 2 (granulocyte-macrophage)	Gm-csf;Gmcsf	U00620.1	136
Rn02134446_s1	Cx3cr1	chemokine (C-X3-C motif) receptor 1	Rbs11	U04808.1	124
Rn00595504_m1	Cxcl9	chemokine (C-X-C motif) ligand 9	Mig;Scyb9	AF462610.1;AF537208.1;BC087594.1	63
Rn00788261_g1	Cxcl11	chemokine (C-X-C motif) ligand 11	SCYB11	BC168725.1;AF537209.1;AY340181.1	67
Rn00570857_s1	Cxcr1	chemokine (C-X-C motif) receptor 1	Il8ra	-	72
Rn03037244_s1	Cxcr6	chemokine (C-X-C motif) receptor 6	-	DQ132631.1	158
Rn00563754_m1	Faslg	Fas ligand (TNF superfamily, member 6)	Apt1Lg1;CD95-L;Fasl;Tnfsf6	U03470.1	101
Rn00821752_g1	Gzmb	Granzyme B-like 2;granzyme B (granzyme 2, cytotoxic T-lymphocyte-associated serine esterase 1)	RNKP-1	BC127475.1;FQ221957.1;FQ228927.1	87
Rn01536933_m1	Hmox1	heme oxygenase (decycling) 1	HEOXG;Heox;Hmox;Ho-1;Ho1;hsp32	FQ228696.1;BC091164.1	94
Rn01437224_m1	Icos	inducible T-cell co-stimulator	Ailim	AB023133.1;AB023134.1	70
Rn00594078_m1	Ifng	interferon gamma	IFNG2	AF010466.1	91
Rn00580432_m1	Il1b	interleukin 1 beta	-	M98820.1;BC091141.1	74

Rn02586400_m1	Il1rn	interleukin 1 receptor antagonist	IL-1ra	CK364274.1;BC070930.1	77
Rn00565865_m1	Il2ra	interleukin 2 receptor, alpha	IL2RAC	M55049.1;FQ228242.1	84
Rn01425162_m1	Il12rb2	interleukin 12 receptor, beta 2	-	AF083329.1	79
Rn00584495_g1	Il18bp	interleukin 18 binding protein	Igifbp	AF154569.1	86
Rn01755623_m1	Il21	interleukin 21	IL-21	DQ387062.1	105
Rn01510484_m1	Il27	interleukin 27	RGD1561420	-	89
Rn01456791_m1	Irf1	interferon regulatory factor 1	-	BC076382.1;M34253.1	66
Rn03993492_g1	Lta	lymphotoxin alpha	Tnfb	-	86
Rn01399572_m1	Nfkb1	nuclear factor of kappa light polypeptide gene enhancer in B-cells 1	EBP-1;NF-kB	-	67
Rn00561646_m1	Nos2	nitric oxide synthase 2, inducible	Nos2a;iNos	D14051.1;AY211532.1;D44591.1;U03699.1;D12520.1;X76881.1;L12562.1;U26686.1;D83661.1	77
Rn01454522_m1	Pdcd1lg2	programmed cell death 1 ligand 2	-	-	67
Rn00569095_m1	Prf1	perforin 1 (pore forming protein)	Cyta;RATCYTA	BC089808.1;M33605.1	87
Rn01483828_m1	Ptgs2	prostaglandin-endoperoxide synthase 2	COX-2;Cox2	AY157736.1;S67722.1;U03389.1;AF233596.1;U04300.1;L25925.1;L20085.1	112
Rn00595838_s1	Socs1	suppressor of cytokine signaling 1	Cish1;Socs-1	AW917497.1	76
Rn01461633_m1	Tbx21	T-box 21	-	-	112
Rn01525859_g1	Tnf	tumor necrosis factor;tumor necrosis factor	RATTNF;TNF-alpha;Tnfa	AJ002278.1;X66539.1;BC107671.1;AF269160.1;AF269159.1	92
Rn00585582_m1	Tnfsf4	tumor necrosis factor (ligand) superfamily, member 4	Ox40l	AF037067.1	101
Rn01764811_m1	Tnfsf8	tumor necrosis factor (ligand) superfamily, member 8	-	-	63
Rn01483793_m1	Tnfsf18	tumor necrosis factor (ligand) superfamily, member 18	-	-	71
Rn01515573_m1	Tnfrsf18	tumor necrosis factor receptor superfamily, member 18	-	BC088265.1	75
Rn01511602_m1	Vegfa	vascular endothelial growth factor A	VEGF-A;VEGF164;VPF;Vegf	AY702972.1;L20913.1;AY033504.1;AY033503.1;AF215726.1;AF215725.1;AY033508.1;AY033507.1;GQ423618.1;AY033506.1;AF222779.1;M32167.1;BC168708.1	95

Fortyfour primers for genes of immunological interest plus three housekeeping genes and one control were purchased from Applied Biosystem. Assay ID, gene symbol(s), gene alias(es), gene bank mRNA, and amplicon length information were provided by the company; specific primers and probes sequences are proprietary information and were not provided by the company.

Supplementary Table 5. Primers for independent rat PCR

Assay ID	Gene Symbol(s)	Gene Name(s)	Gene Alias(es)	GenBank mRNA(s)	Amplicon Length
Rn01775763_g1	Gapdh	glyceraldehyde-3-phosphate dehydrogenase	BARS-38;Gapd	AB017801.1;M17701.1;BC087743.1;M29341.1;X02231.1;M11561.1;BC059110.1	174
Rn00566655_m1	Gusb	glucuronidase, beta	Ac2-223;Gus-s	M13962.1;Y00717.1	63
Rn00820645_g1	Rps29	ribosomal protein S29	-	FQ223975.1;FQ221523.1;FQ216880.1;FQ228434.1;FQ224820.1;FQ209501.1;FQ221661.1;FQ217213.1;FQ223378.1;FQ222368.1;FQ217140.1;FQ222293.1;X59051.1;FQ222143.1;FQ223222.1;FQ221457.1;FQ224661.1;FQ224465.1;FQ221640.1;FQ221587.1;FQ223289.1;BC058150.1;FQ221360.1;FQ223885.1;FQ218769.1;FQ211740.1;FQ221707.1;FQ228730.1;FQ224325.1;FQ222711.1;FQ221030.1;FQ222566.1;FQ223071.1;FQ224400.1;FQ221875.1;FQ210507.1;FQ218037.1;FQ224476.1;FQ221307.1;FQ218006.1;FQ222937.1;FQ221298.1;FQ221942.1;FQ221251.1	73
Rn00579590_m1	Ccl5	chemokine (C-C motif) ligand 5	Rantes;Scya5	U06436.1;EF122007.1;BC058138.1;EF121972.1	80
Rn01637698_s1	Ccr2	chemokine (C-C motif) receptor 2	-	-	103
Rn02134292_s1	Ccr3	chemokine (C-C motif) receptor 3	Cmkbr3	Y13400.1	80
Rn02132969_s1	Ccr5	chemokine (C-C motif) receptor 5	Ckr5;Cmkbr5	BC078756.1	81
Rn01456850_m1	Csf2	colony stimulating factor 2 (granulocyte-macrophage)	Gm-csf;Gmcsf	U00620.1	136
Rn00595504_m1	Cxcl9	chemokine (C-X-C motif) ligand 9	Mig;Scyb9	AF462610.1;AF537208.1;BC087594.1	63
Rn00788261_g1	Cxcl11	chemokine (C-X-C motif) ligand 11	SCYB11	BC168725.1;AF537209.1;AY340181.1	67
Rn03037244_s1	Cxcr6	chemokine (C-X-C motif) receptor 6	-	DQ132631.1	158
Rn00563754_m1	Faslg	Fas ligand (TNF superfamily, member 6)	Apt1Lg1;CD95-L;Fasl;Tnfsf6	U03470.1	101
Rn00821752_g1	Gzmb	Granzyme B-like 2;granzyme B (granzyme 2, cytotoxic T-lymphocyte-associated serine esterase 1)	RNKP-1	BC127475.1;FQ221957.1;FQ228927.1	87
Rn01536933_m1	Hmox1	heme oxygenase (decycling) 1	HEOXG;Heox;Hmox;Ho-1;Ho1;hsp32	FQ228696.1;BC091164.1	94
Rn00594078_m1	Ifng	interferon gamma	IFNG2	AF010466.1	91
Rn00565865_m1	Il2ra	interleukin 2 receptor, alpha	IL2RAC	M55049.1;FQ228242.1	84
Rn01425162_m1	Il12rb2	interleukin 12 receptor, beta 2	-	AF083329.1	79
Rn00584495_g1	Il18bp	interleukin 18 binding protein	Igifbp	AF154569.1	86
Rn01755623_m1	Il21	interleukin 21	IL-21	DQ387062.1	105
Rn03993492_g1	Lta	lymphotoxin alpha	Tnfb	-	86
Rn00561646_m1	Nos2	nitric oxide synthase 2, inducible	Nos2a;iNos	D14051.1;AY211532.1;D44591.1;U03699.1;D12520.1;X76881.1;L12562.1;U26686.1;D83661.1	77
Rn01525859_g1	Tnf	tumor necrosis factor;tumor necrosis factor	RATTNF;TNF-alpha;Tnfa	AJ002278.1;X66539.1;BC107671.1;AF269160.1;AF269159.1	92
Rn00595838_s1	Socs1	suppressor of cytokine signaling 1	Cish1;Socs-1	AW917497.1	76
Rn01461633_m1	Tbx21	T-box 21	-	-	112

Independent primers for genes of immunological interest plus three housekeeping genes were purchased from Applied Biosystem. Assay ID, gene symbol(s), gene alias(es), gene bank mRNA, and amplicon length information were provided by the company; specific primers and probes sequences are proprietary information and were not provided by the company.
