# Supplementary material for: Working correlates of protection predict SchuS4-derived-vaccine candidates with improved efficacy against an intracellular bacterium, Francisella tularensis
Source: NPJ Vaccines. 2022 Aug 17;7:95. doi: 10.1038/s41541-022-00506-9 (PMC9385090; doi:10.1038/s41541-022-00506-9)
Supplement: Supplementary file 2 — REPORTING SUMMARY [file 41541_2022_506_MOESM2_ESM.pdf]

nature portfolio

Corresponding author(s): DBPR NPJVACCINES-01566R3

Last updated by author(s): May 30, 2022

Reporting Summary

Nature Portfolio wishes to improve the reproducibility of the work that we publish. This form provides structure for consistency and transparency in reporting. For further information on Nature Portfolio policies, see our Editorial Policies and the Editorial Policy Checklist.

Statistics

For all statistical analyses, confirm that the following items are present in the figure legend, table legend, main text, or Methods section.

n/a	Confirmed	
☐	☒	The exact sample size (*n*) for each experimental group/condition, given as a discrete number and unit of measurement
☐	☒	A statement on whether measurements were taken from distinct samples or whether the same sample was measured repeatedly
☐	☒	The statistical test(s) used AND whether they are one- or two-sided<br>*Only common tests should be described solely by name; describe more complex techniques in the Methods section.*
☐	☒	A description of all covariates tested
☐	☒	A description of any assumptions or corrections, such as tests of normality and adjustment for multiple comparisons
☐	☒	A full description of the statistical parameters including central tendency (e.g. means) or other basic estimates (e.g. regression coefficient) AND variation (e.g. standard deviation) or associated estimates of uncertainty (e.g. confidence intervals)
☒	☐	For null hypothesis testing, the test statistic (e.g. *F*, *t*, *r*) with confidence intervals, effect sizes, degrees of freedom and *P* value noted<br>*Give P values as exact values whenever suitable.*
☒	☐	For Bayesian analysis, information on the choice of priors and Markov chain Monte Carlo settings
☒	☐	For hierarchical and complex designs, identification of the appropriate level for tests and full reporting of outcomes
☒	☐	Estimates of effect sizes (e.g. Cohen's *d*, Pearson's *r*), indicating how they were calculated

Our web collection on statistics for biologists contains articles on many of the points above.

Software and code

Policy information about availability of computer code

Data collection	Softmax Pro Version 5.4<br>Diva, Version 8.0.3<br>ViiA7 Software, QuantStudio, Version 1.6.1
Data analysis	Excel, Version 2102<br>GraphPad Prism Version, 8.2.1<br>The software programming language R Version 4.0.2. R packages “nnet”, “leaps”, and “performance” were loaded into R to implement multinomial regression, best subsets regression, and the calculation of McFadden’s R2, respectively.<br>FlowJo, Version 10.7.1

For manuscripts utilizing custom algorithms or software that are central to the research but not yet described in published literature, software must be made available to editors and reviewers. We strongly encourage code deposition in a community repository (e.g. GitHub). See the Nature Portfolio guidelines for submitting code & software for further information.

Data

Policy information about availability of data

All manuscripts must include a data availability statement. This statement should provide the following information, where applicable:

- Accession codes, unique identifiers, or web links for publicly available datasets
- A description of any restrictions on data availability
- For clinical datasets or third party data, please ensure that the statement adheres to our policy

Data generated or analyzed during this study are included in this published article and its supplementary information files; data not shown are available from the corresponding author on request

Field-specific reporting

Please select the one below that is the best fit for your research. If you are not sure, read the appropriate sections before making your selection.

☒ Life sciences ☐ Behavioural & social sciences ☐ Ecological, evolutionary & environmental sciences

For a reference copy of the document with all sections, see nature.com/documents/nr-reporting-summary-flat.pdf

Life sciences study design

All studies must disclose on these points even when the disclosure is negative.

Sample size	For vitro studies, 3 rats or 7 mice / vaccine group, were used to obtain an adequate amount of PBLs to perform the co-culture assays. For survival studies, 3-5 mice or 3-7 rats were used for each replicate.
Data exclusions	No data were excluded from the analyses.
Replication	The in vitro studies were repeated 4-5 times. The survival studies were repeated 3-5 times.
Randomization	The studies involved inbred animals and the animals were not randomized.
Blinding	The study was not blinded.

Reporting for specific materials, systems and methods

We require information from authors about some types of materials, experimental systems and methods used in many studies. Here, indicate whether each material, system or method listed is relevant to your study. If you are not sure if a list item applies to your research, read the appropriate section before selecting a response.

Materials & experimental systems

n/a	Involved in the study	
☐	☒	Antibodies
☒	☐	Eukaryotic cell lines
☒	☐	Palaeontology and archaeology
☐	☒	Animals and other organisms
☒	☐	Human research participants
☒	☐	Clinical data
☒	☐	Dual use research of concern

Methods

n/a	Involved in the study	
☒	☐	ChIP-seq
☐	☒	Flow cytometry
☒	☐	MRI-based neuroimaging

Antibodies

Antibodies used

Flow Cytometry:
anti-B220 (clone RA3-6B2), Biolegend, Cat #103227, Lot #B150120; BD Cat #557957, Lot #38765.
anti-CD19 (clone 1D3), BD, Cat #557398, Lot #5138957; BD, Cat #557399, Lot #5141840.
anti-TCRβ (clone H57-597), BD, Cat #553171, Lot #3228998; BD, Cat #553174, Lot #38374.
anti-CD4 (clone RM4-5), BD, Cat #557956, Lot #14405; BD, Cat #553051, Lot #28458.
anti-CD8 (H35-17.2), BD, Cat #550798, Lot #28268.
anti-NK1.1 (clone PK136), Biolegend, Cat #108740, Lot #B210016.
anti-CD11b (clone M1/70), BD, Cat #552850, Lot #5126843.
anti-Gr-1 (clone RB6-8C5), BD, Cat #553129, Lot #59514.
anti-CD11c (cloneHL3), Biolegend, Cat #117329, Lot #B209019.
anti-CD45 (clone 30-F11), Biolegend, Cat #103130, Lot #B214531.

Rat IFN-gamma ELISA:
Capture Ab, Biolegend, Cat # 507801, Lot #B207530
Standard Ab, Biolegend, Cat # 580009, Lot #B209102
Detection Ab, Biolegend, Cat #518803, Lot #B342999
Rat Serum ELISA: Anti-rat IgG-HRP, Southern Biotech, Cat #3030-05, Lot #K0810-YO6E
Mouse IFN-gamma ELISA:
Capture Ab, BD, Cat #551216, Lot #9150938
Standard Ab, BD, Cat # 554587, Lot #9049603
Detection Ab, BD Cat # 554410, Lot #9176274

Validation

All were commercial antibodies, validated by the vendors.

Animals and other organisms

Policy information about studies involving animals; ARRIVE guidelines recommended for reporting animal research

Laboratory animals	6-12 week old C57BL/6J mice. 6-11 week old female Fischer 344 rats.
Wild animals	The study did not involve wild animals
Field-collected samples	The study did not involve field-collected samples.
Ethics oversight	Animal Care and Use Committees of CBER, FDA and University of New Mexico

Note that full information on the approval of the study protocol must also be provided in the manuscript.

Flow Cytometry

Plots

Confirm that:

☒ The axis labels state the marker and fluorochrome used (e.g. CD4-FITC).

☒ The axis scales are clearly visible. Include numbers along axes only for bottom left plot of group (a 'group' is an analysis of identical markers).

☒ All plots are contour plots with outliers or pseudocolor plots.

☒ A numerical value for number of cells or percentage (with statistics) is provided.

Methodology

Sample preparation	Single cell suspensions were prepared from total splenocytes and PBLs, and from cells recovered from co-cultures. Cells were incubated with anti-CD16/CD32 and stained with Live/Dead staining kit. Cells were then washed in flow cytometry buffer and stained for cell surface markers. Antibody concentrations were previously optimized for use in multi-color staining protocols as required, using appropriate fluorochrome-labeled isotype matched control antibodies.
Instrument	LSR-II, BD, not longer available.<br>LSR-Fortessa, BD, special order research product with 5 lasers (UV 355nm/Violet 405nm/Blue 488nm/Light-Green 552nm/ Red 640nm)
Software	Diva (BD), Version 8.0.3<br>FlowJo (Tree Star, Inc), Version 10.7.1
Cell population abundance	A minimum of ten thousand total events were counted and acquired.
Gating strategy	Cells were initially gated for Live CD45+ cells. Then, fragments (FSC-A vs. SSC-A) and aggregates (FSC-A vs. FSC-H) were excluded. Cells were then gated for B220 and CD19 to identify B cells, for TCRb and CD4 and TCRb and CD8 to identify T cells. Non B or T cells were gated for Gr1 and Cd11b to identify neutrophils. Non-neutrophils were gated for Cd11b and CD11c to identify DC and macrophages. Finally, the non-DC or macrophages were gated for NK1.1 to identify natural killer.

☒ Tick this box to confirm that a figure exemplifying the gating strategy is provided in the Supplementary Information.
